# Cell shape and division septa positioning in filamentous *Streptomyces* require a functional cell wall glycopolymer ligase CglA

**DOI:** 10.1128/mbio.01492-24

**Published:** 2024-09-09

**Authors:** Sukanya Bhowmick, Ruth P. Viveros, Andreas Latoscha, Fabian M. Commichau, Christoph Wrede, Mahmoud M. Al-Bassam, Natalia Tschowri

**Affiliations:** 1 Institute of Microbiology, Leibniz Universität Hannover, Hannover, Germany; 2 Institute of Biology/Microbiology, Humboldt-Universität zu Berlin, Berlin, Germany; 3 Institute of Biology, FG Molecular Microbiology 190 h, Universität Hohenheim, Stuttgart, Germany; 4 Institute of Functional and Applied Anatomy, Research Core Unit Electron Microscopy, Hannover Medical School, Hannover, Germany; 5 Department of Pediatrics, University of California, San Diego, California, USA; Duke University School of Medicine, Durham, North Carolina, USA

**Keywords:** LCP-domain, FtsZ, sporulation, *Streptomyces*, cell wall glycopolymers

## Abstract

The cell wall of monoderm bacteria consists of peptidoglycan and glycopolymers in roughly equal proportions and is crucial for cellular integrity, cell shape, and bacterial vitality. Despite the immense value of *Streptomyces* in biotechnology and medicine as antibiotic producers, we know very little about their cell wall biogenesis, composition, and functions. Here, we have identified the LCP-LytR_C domain protein CglA (Vnz_13690) as a key glycopolymer ligase*,* which specifically localizes in zones of cell wall biosynthesis in *S. venezuelae*. Reduced amount of glycopolymers in the *cglA* mutant results in enlarged vegetative hyphae and failures in FtsZ-rings formation and positioning. Consequently, division septa are misplaced leading to the formation of aberrant cell compartments, misshaped spores, and reduced cell vitality. In addition, we report our discovery that c-di-AMP signaling and decoration of the cell wall with glycopolymers are physiologically linked in *Streptomyces* since the deletion of *cglA* restores growth of the *S. venezuelae disA* mutant at high salt. Altogether, we have identified and characterized CglA as a novel component of cell wall biogenesis in *Streptomyces*, which is required for cell shape maintenance and cellular vitality in filamentous, multicellular bacteria.

**IMPORTANCE:**

*Streptomyces* are our key producers of antibitiotics and other bioactive molecules and are, therefore, of high value for medicine and biotechnology. They proliferate by apical extension and branching of hyphae and undergo complex cell differentiation from filaments to spores during their life cycle. For both, growth and sporulation, coordinated cell wall biogenesis is crucial. However, our knowledge about cell wall biosynthesis, functions, and architecture in *Streptomyces* and in other Actinomycetota is still very limited. Here, we identify CglA as the key enzyme needed for the attachment of glycopolymers to the cell wall of *S. venezuelae*. We demonstrate that defects in the cell wall glycopolymer content result in loss of cell shape in these filamentous bacteria and show that division-competent FtsZ-rings cannot assemble properly and fail to be positioned correctly. As a consequence, cell septa placement is disturbed leading to the formation of misshaped spores with reduced viability.

## INTRODUCTION

The bacterial cell envelope is the first barrier against environmental stresses and is crucial for cellular integrity, shape, and size. The core components of the cell envelope in monoderm (Gram-positive) bacteria represent multiple layers of peptidoglycan (PG) and teichoic acids, both present in roughly equal proportions (1, 2). PG consists of linear glycan strands composed of alternating *N*-acetylglucosamine and *N*-acetylmuramic acid cross-linked by short peptides to form a murein sacculus, which is central for providing mechanical strength to withstand the turgor pressure and serves as a scaffold for numerous proteins and glycopolymers (3). Teichoic acids are a diverse class of glycopolymers containing phosphodiester-linked polyol repeat units, such as poly glycerol-3-phosphate or poly ribitol-5-phosphate, which are often decorated with *N*-acetylhexosamine residues and D-alanine, respectively (1). These polymers can be grouped into two types: lipoteichoic acids (LTAs), which are anchored to the cytoplasmic membrane via a glycolipid, and wall teichoic acids (WTAs), which are linked via a phosphodiester bond to the C6 hydroxyl of *N*-acetylmuramic acid of peptidoglycan (4).

WTAs are key determinants of virulence and antibiotic resistance in many pathogens (5). Therefore, inhibition of WTA biosynthesis is a powerful therapeutic approach and has been in focus of screening campaigns to find novel antimicrobials (6, 7). Beyond their roles as virulence factors required for host colonization (8), WTAs fulfill important functions in cell division by contributing to the proper localization of autolysins to the division septum for PG breakdown (9, 10), in peptidoglycan biosynthesis by affecting spatiotemporal localization of penicillin-binding proteins (11), in stress tolerance (12), and in phage binding (13).

Glycopolymer ligases of the LytR-CpsA-Psr (LCP) protein family catalyze the covalent attachment of WTAs to PG (9, 14–17). In contrast to diderm (Gram-negative) bacteria, virtually all monoderm bacteria possess LCP proteins, and most of them have multiple copies of these proteins (18). However, while Bacillota can have up to 6 copies, the situation is far more complex in most Actinomycetota, for example, *Streptomyces coelicolor* contains 11 proteins of the LCP type (18, 19). Moreover, in Actinomycetota, the LCP domain can occur separately, but also in combination with the LytR_C domain of yet unknown function, often located at the C-terminus of proteins (20).

Streptomycetes are important members of the soil microbiome which are renowned for their contribution to the reduction of infections and other diseases due to their immense potential to synthesize diverse antibiotics, anticancer compounds, immunosuppressants, and antiviral substances (21, 22). During growth, these bacteria undergo a complex transition from multicellular vegetative hyphae to unicellular spores. They gain biomass by apical extension of their filaments and by initiating new branches behind the tip (23). Apical growth is directed by the cytoskeletal-like coiled-coil protein DivIVA that together with other proteins, such as Scy and FilP, forms the polarisome, which is needed for proper localization of peptidoglycan synthases, hydrolases, and other proteins involved in cell wall assembly and establishment of cell polarity (24, 25). For reproduction, Streptomycetes initiate FtsZ-driven division of sporogenic hyphae into dozens of pre-spore compartments. This involves the formation of ladder-like arrays of multiple FtsZ-rings along the filament resulting in the emergence of regularly shaped and sized spores after constriction of the FtsZ-rings and cell-cell separation (26, 27).

In soil, Streptomycetes and other microorganisms are often exposed to changes in external osmotic conditions caused by rainfall or drought and have evolved diverse strategies for the adaptation to variations in osmolality (28). The nucleotide second messenger cyclic di-3′,5′-adenosine monophosphate (c-di-AMP) is a key regulator of osmotic stress responses in bacteria by controlling ion and osmolyte transport for the maintenance of cellular osmotic homeostasis and turgor (29). In *Streptomyces venezuelae*, the DAC-domain containing diadenylate cyclase DisA produces c-di-AMP out of adenosine triphosphate (ATP), while the phosphodiesterase AtaC has the opposite activity and degrades c-di-AMP to adenosine monophosphate (AMP) via 5′-phosphoadenylate-(3′−5′)-adenosine pApA. c-di-AMP enables *Streptomyces* to survive osmotic stress since a strain defective in c-di-AMP biosynthesis due to deletion or inactivation of *disA* has a growth defect on nutrient agar supplemented with high salt (30). Up to now, two direct c-di-AMP targets have been identified in *Streptomyces*: the *ydaO*-like riboswitch in the 5′-UTR of the *rpfA* gene, coding for the cell wall hydrolase RpfA (31), and the RCK_C (regulator of conductance of K^+^) domain of the CpeA protein, which is likely a regulatory subunit of a predicted ion transport system (30, 32).

What exactly compromises *Streptomyces* viability at high salt concentrations, when c-di-AMP levels are too low, is unknown. In this study, we addressed this question by screening for suppressor mutants and found that inactivation of the LCP-LytR_C-domain containing protein Vnz_13690 (CglA; for cell wall glycopolymer ligase A) enables *S. venezuelae disA* mutant to grow at high NaCl concentration on nutrient agar. We demonstrate that deletion of *cglA* leads to reduced glycopolymer content in the cell wall fraction and consequently to failures in cell shape maintenance, FtsZ-rings positioning, and septa placement resulting in reduced cell viability and developmental defects.

## RESULTS

### Deletion of *cglA* restores resistance to ionic osmotic stress in *S. venezuelae* ∆*disA*

**Fig 1:**
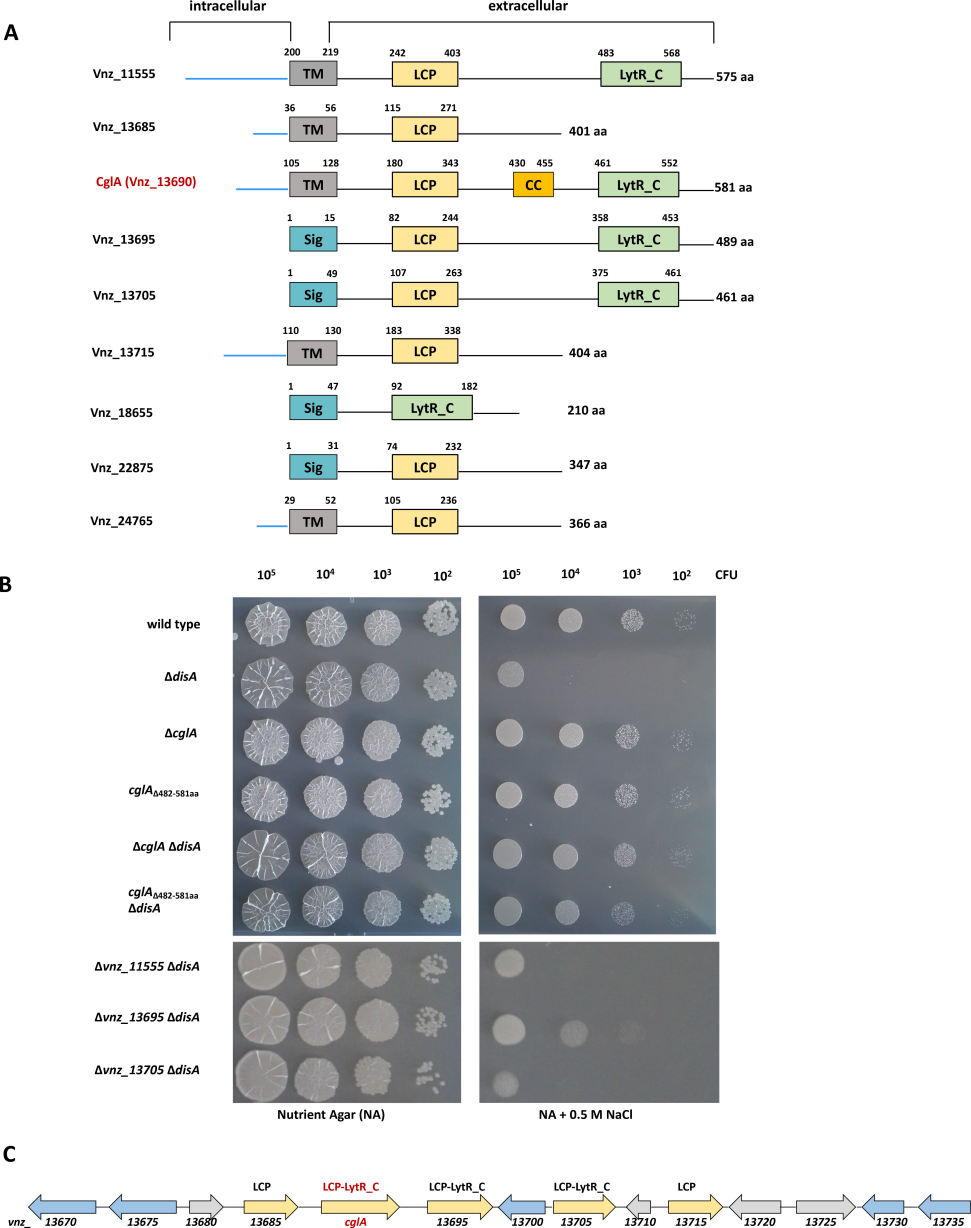
LCP- and LytR_C-domain proteins in *S. venezuelae* and their impact on salt sensitivity of the *disA* mutant. (**A**) Domain organization of LCP- and LytR_C-domain proteins as predicted using UniProt (33) and TMHMM 2.0 (34). The N-terminal cytoplasmic portion of the proteins is visualized as a blue line. LCP, LytR-CpsA-Psr; TM, transmembrane; Sig, signal peptide; CC, coiled-coil region. (**B**) Deletion of *cglA* or *vnz_13695* suppresses salt sensitivity of the *disA* mutant. Serial dilutions of spores of indicated strains were spotted on nutrient agar (NA) with or without added 0.5 M NaCl and grown at 30°C for 2 days. (**C**) *cglA* is located in a genetic hot spot for genes encoding putative cell wall modifying enzymes (yellow and blue arrows). Yellow: genes encoding proteins containing an LCP and/or LytR_C domain (see also panel **A**). Blue: *vnz_13670* (putative *N*-acetylglucosamine-1-phosphate uridyltransferase or glucosamine-1-phosphate *N*-acetyltransferase); *vnz_13675* (putative *N*-acetylmuramoyl-l-alanine amidase); *vnz_13700* (putative glycosyl transferase); *vnz_13730* and *vnz_13735* (putative dipeptidases).

Depletion of c-di-AMP due to the inactivation of *disA* renders *S. venezuelae* highly susceptible to ionic osmostress (30). To understand which cellular functions are directly or indirectly linked to c-di-AMP signaling pathways in *Streptomyces*, we used a genetic approach by screening for suppressors that restore osmoresistance of the *S. venezuelae disA* mutant. We cultivated the mutant on nutrient agar containing 0.5 M NaCl and isolated 23 single colonies which appeared after 3.5 days of incubation and showed improved growth despite the presence of high salt. We isolated spores from these 23 candidate strains and excluded all clones that had growth defects on nutrient agar without added salt from further analyses. Using whole-genome sequencing, we analyzed five clones carrying the *disA* deletion and showing a stable osmoresistant phenotype after a subsequent round of spore collection. With respect to mutations within open reading frames, we found several alterations in the sequenced genomes (Table S1). Of these, the G1446A mutation in the *cglA* (*vnz_13690*) gene in the *disA* suppressor strain 1 (∆*disA*-S1) caught our attention. This allele exchange alters the tryptophan at position 482 to the TGA stop codon in the LCP-LytR_C-domain protein CglA (Fig. 1A), resulting in a truncated protein variant missing 99 amino acids in the C-terminus that comprise the LytR_C domain (CglA_∆482–581aa_).

To confirm that truncation or inactivation of CglA rescues the salt-sensitive phenotype of ∆*disA*, we applied λ RED-mediated recombineering to generate a *cglA*_∆482–581aa_ mutant and a *cglA* deletion strain by replacing the relevant gene regions with the hygromycin resistance cassette (35). We then applied SV1 phage-mediated transduction to create *cglA::hyg disA::apr* and *cglA*_∆482–581aa_*::hyg disA::apr* double mutants. As shown in Fig. 1B, both, deletion of the entire *cglA* gene and of the gene region covering just the LytR_C domain in CglA, bypasses the need for c-di-AMP produced by *disA* for the adaptation to high external salt concentration. Of note, single *S. venezuelae cglA* and *cglA*_∆482–581aa_ mutants grew as the wild type on nutrient agar (NA) containing added 0.5 M NaCl (Fig. 1B).

*S. venezuelae* has four genes encoding proteins with an LCP-LytR_C domain architecture. Apart from CglA, these are Vnz_11555, Vnz_13695, and Vnz_13705. In addition, four genes encode for proteins with a single LCP domain (*vnz_13685*, *vnz_13715*, *vnz_22875*, and *vnz_24765*), and Vnz_18655 represents a stand-alone LytR_C domain protein (Fig. 1A). Five of these genes (*vnz_13685*, *cglA*, *vnz_13695*, *vnz_13705,* and *vnz_13715*) are located within one gene cluster but do not seem to form an operon (Fig. 1C). We wondered whether inactivation of any of the other LCP-LytR_C-domain containing protein can suppress salt sensitivity of the *disA* mutant similar to *cglA*. Thus, we created single *S. venezuelae vnz_11555::hyg*, *vnz_13695::hyg,* and *vnz_13705::hyg* mutants as well as double mutants in combination with *disA::apr*. As shown in Fig. 1B, deletion of *vnz_13695* could also restore salt resistance of the *disA* mutant to a minor extent, while single mutants grew similar to the wild type on nutrient agar containing extra 0.5 M NaCl (Fig. S1).

### CglA is required for regular sporulation in *S. venezuelae*

**Fig 2:**
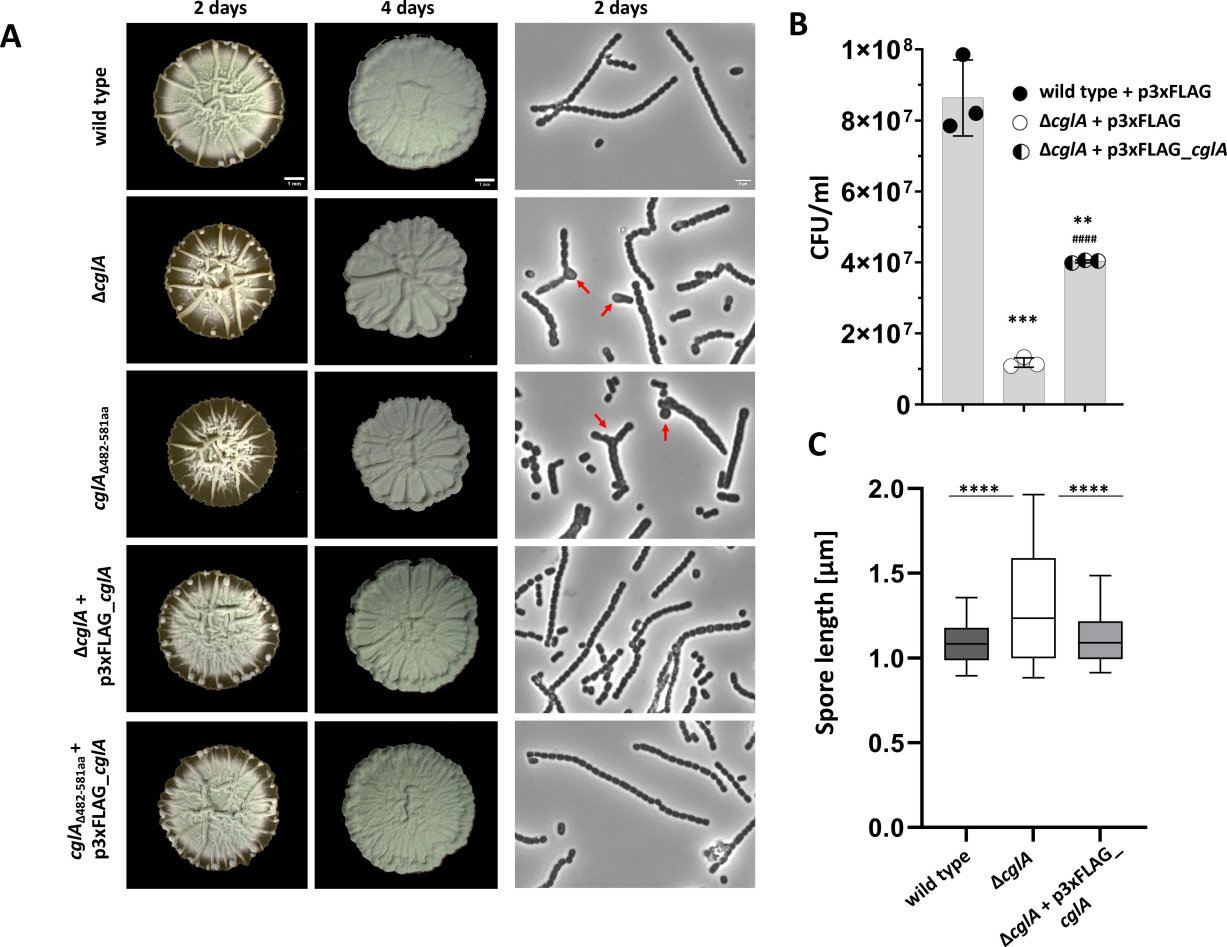
Inactivation of CglA leads to sporulation defects in *S. venezuelae*. (**A**) Macrocolonies of *S. venezuelae* wild type, *cglA::hyg* mutant, strain expressing a truncated *cglA*_∆482–581aa_ variant and of respective strains carrying *cglA* controlled by the native promoter on the integrative p3xFLAG vector for complementation. Twelve microliters of 2 × 10^5^ CFU/µL spores was spotted on MYM agar and grown at 30°C for either 2 (left) or 4 days (middle). Scale bar: 1 mm. Right lane: images showing coverslip imprints of the indicated strains taken from the surface of macrocolonies after 2 days of incubation. Red arrows point to deformed spores formed by the *cglA* mutants. Scale bar: 2 µm. (**B**) Sporulation efficiency (in CFU/mL) of the *S. venezuelae* wild type and *cglA* mutant (both carrying the empty p3xFLAG vector) and of the mutant strain expressing *cglA* from p3xFLAG. Four macrocolonies for each strain were harvested and transferred into 2 mL 20% (vol/vol) glycerol. After vortexing, the suspension was centrifuged for 2 min at 2,000 rpm and 1 mL of the supernatant containing the spores was filtered using a 5-µm filter. Filtrates containing the spores were diluted, and appropriate dilutions were plated on LB agar. Colonies were counted after 2 days of incubation at 30°C. Data obtained from three independent biological replicates are plotted as mean with error bars representing standard deviation that were statistically analyzed using unpaired *t*-test, (***P < 0.001, **P < 0.01, ^####^*P* < 0.0001). Comparison with wild type+p3xFLAG (*) and with ∆*cglA*+p3xFLAG (#). (**C**) Cell length of spores formed by *S. venezuelae* wild type, *cglA* mutant, and ∆*cglA*+p3xFLAG-*cglA* strain was measured (*n* = 347 for each strain). Box plots are plotted with a line at the median and whiskers represent 10–90 percentile of the plotted data set. Kruskal-Wallis with Dunn’s multiple comparison tests (****P value < 0.0001) was performed to show statistical significance.

On solid sporulation medium, distinct stages of *Streptomyces* development are characterized by a particular colony morphology. *S. venezuelae* colonies appear white on maltose-yeast extract-malt extract (MYM) agar medium when the bacteria erect aerial hyphae to initiate sporulation and turn green when spores mature due to the accumulation of a polyketide pigment (36). We found that both, the *cglA::hyg* and the *cglA*_∆482-581aa_*::hyg,* mutant have an indistinguishable developmental phenotype. While a wild-type colony becomes green after 2 days of growth on MYM agar, both mutants form white colonies after the same incubation time (Fig. 2A, left row) but become green after prolonged incubation of 4 days (Fig. 2A, middle row). Of note, the deletion of *cglA* does not affect *S. venezuelae* growth in liquid MYM, as determined by measurements of optical density along the growth cycle (Fig. S2A). The developmental defect of the two mutants could be complemented by the expression of *cglA* controlled by the native promoter from the p3xFLAG (Apr^R^) vector (37), a derivative of pIJ10770 that integrates into the ΦBT1 attachment site (38) (Fig. 2A). However, expressing *cglA* versions that either lack the intracellular portion of the protein (amino acids 2–94; CglA_∆IP), the LCP domain (amino acids 180–343; CglA_∆LCP) or the coiled-coil region (amino acids 430–455; CglA_∆CC) from the same vector, did not restore development of the *cglA* mutant (Fig. S3). Strikingly, out of the four LCP-LytR_C-domain proteins in *S. venezuelae*, only CglA has a non-redundant function in cell differentiation since deletion of either *vnz_11555*, *vnz_13695,* or *vnz_13705* had no detectable consequences on development (Fig. S4). To understand the expression profiles of genes encoding LCP- or LytR_C-domain proteins, we have extracted transcription data for the relevant genes from time-course microarray experiments performed in the study by Bibb et al. (39), as described in Al-Bassam et al. (37). The expression data show that within the analyzed set, *cglA* belongs to the most highly expressed genes in liquid MYM. However, after 16 h of growth, transcripts of *vnz_13685* and *vnz_18655* are most abundant (Fig. S5).

To quantify the sporulation defect of the *cglA::hyg* mutant, we have harvested spores formed by the respective strains carrying either the empty p3xFLAG vector or p3xFLAG-*cglA* after 48 h of growth on MYM agar and determined colony-forming units (CFU). This analysis confirmed that the deletion of *cglA* results in compromised sporulation. As such, the sporulation efficiency of the mutant was determined to be in average about 13% (1.18 × 10^7^ CFU/mL) when compared to the wild-type set to 100% (8.63 × 10^7^ CFU/mL). Notably, the expression of *cglA* from the integrative p3xFLAG vector restored the sporulation efficiency of the mutant to 46.6% (4.03 × 10^7^ CFU/mL) (Fig. 2B). The *cglA* mutant formed less viable spores in liquid MYM too, demonstrating that the sporulation defect of the mutant is not confined to growth on solid medium (Fig. S2B). Furthermore, coverslip imprints from the upper layer of macrocolonies revealed that besides typical rod-shaped spores, the mutant produced many spores of irregular shape and size (mini, round, asymmetric; see red arrows in Fig. 2A). We have measured the length of about 347 spores for each, the wild type, the *cglA* mutant, and the complemented strain. The average length of wild-type spores was calculated to be 1.114 ± 0.21 µm, while the average length of spores formed by the mutant increased to 1.335 ± 0.45 µm (Fig. 2C). The expression of *cglA* from p3xFLAG in the mutant led to a reduction of the spore length similar to the length of spores formed by the wild type (1.143 ± 0.26 µm).

Overall, these data suggest that CglA is important for efficient sporulation and phenotypic homogeneity of spores formed by *S. venezuelae* and that all the here studied fragments of CglA, i.e., the intracellular portion, the LCP domain, the coiled-coil region, and the LytR_C domain contribute to protein function or its stability.

### CglA contributes to correct FtsZ positioning and septa placement

**Fig 3:**
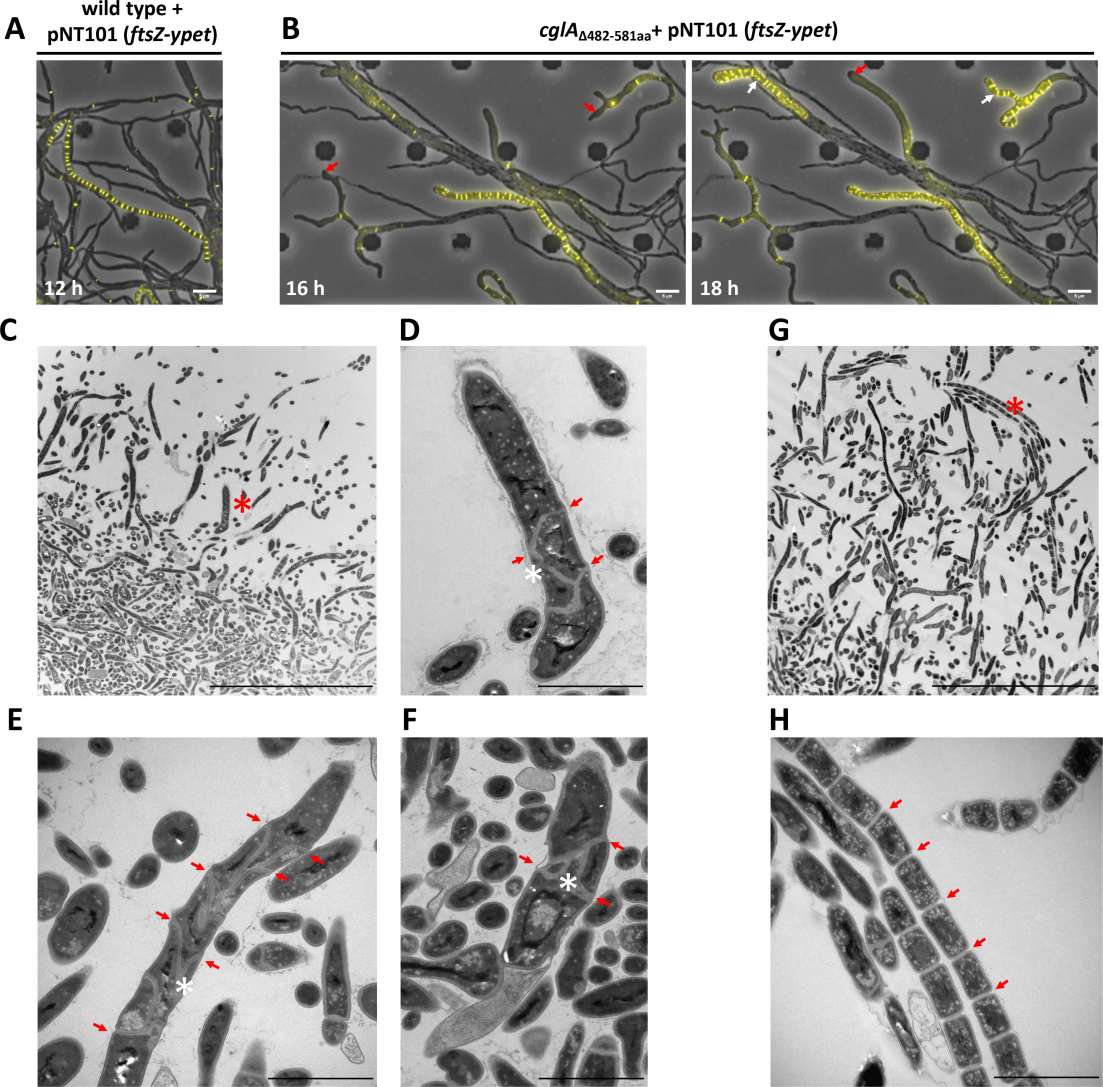
The *cglA* mutant forms hyphae with anomalous morphology and shows failures in FtsZ-ring positioning and septa placement. (**A**) Subcellular localization of fluorescently labeled FtsZ in *S. venezuelae* wild type expressing *ftsZ-ypet* from the integrative pNT101 vector. Processed snapshots from time-lapse imaging experiments show the formation of regularly spaced FtsZ-YPet-rings in a sporogenic hypha. (**B**) The *cglA*_∆482–581aa_ mutant carrying pNT101 contains expanded tips and hyphae (red arrows) in which the formation and localization of FtsZ-YPet-rings is disturbed (white arrows). Scale bar: 5 µm. (**C–H**) TEM analysis of *S. venezuelae* colonies grown for 36 h on MYM agar. Overview (C; region of magnification marked by red asterisk) and close-up (**D–F**) of the *cglA* mutant with malformed septa highlighted with red arrows. Overview (**G**) and magnification (**H**) of *S. venezuelae* wild type showing spore chains with regularly arranged septa. Cell compartments free of DNA are marked with a white asterisk (**D–F**). Scale bars: C, G: 20 µm; D, E, F, H: 2 µm.

In Streptomycetes, the formation and localization of division-competent FtsZ-rings in the sporogenic hyphae determines the synthesis of septal peptidoglycan and affects the size and morphology of spores (40–42). Thus, we hypothesized that the spatial localization of FtsZ might be disturbed upon the inactivation of *cglA*. To address this hypothesis, we introduced an *ftsZ-ypet* fusion controlled by the native *ftsZ* promoter on the pNT101 plasmid into the *cglA*_∆482–581aa_ mutant and wild-type *S. venezuelae* and followed the formation of Z-rings using time-lapse fluorescence microscopy. As expected, we detected the assembly of Z-rings into a characteristic ladder-like pattern in sporogenic hyphae of the wild type (Fig. 3A; Movie S1). However, live-cell imaging revealed that the *cglA*_∆482–581aa_ mutant contained many hyphae with aberrant morphology (Fig. 3B; Movie S2). These hyphae contained swollen hyphal tips (red arrows in Fig. 3B) and increased width. While the width of wild-type hyphae was calculated to be 0.98 ± 0.099 µm, the width of the anomalous hyphae formed by the mutant was determined to be about twofold increased (2.14 ± 0.420 µm). Consequently, upon sporulation initiation of those hyphae, assembly of FtsZ-YPet into ring structures failed, Z-ladders were not properly formed, and spacing between individual FtsZ-YPet-rings was highly irregular (white arrows in Fig. 3B). In line with the observed failures in FtsZ-YPet positioning, transmission electron microscopy (TEM) revealed failures in cell septa placement in the *cglA* mutant, explaining the formation of misshaped spores. Moreover, based on TEM imaging, some of the irregular cell compartments in the mutant appeared to be free of DNA, likely causing a reduction in the formation of viable spores (Fig. 3C through H). We excluded that FtsZ levels are affected by the deletion of *cglA* (Fig. S6) and concluded that CglA plays a crucial role in the correct positioning of FtsZ and cell septa placement during sporulation initiation in *Streptomyces*.

### YPet-CglA localizes at growing hyphal tips and branching zones

**Fig 4:**
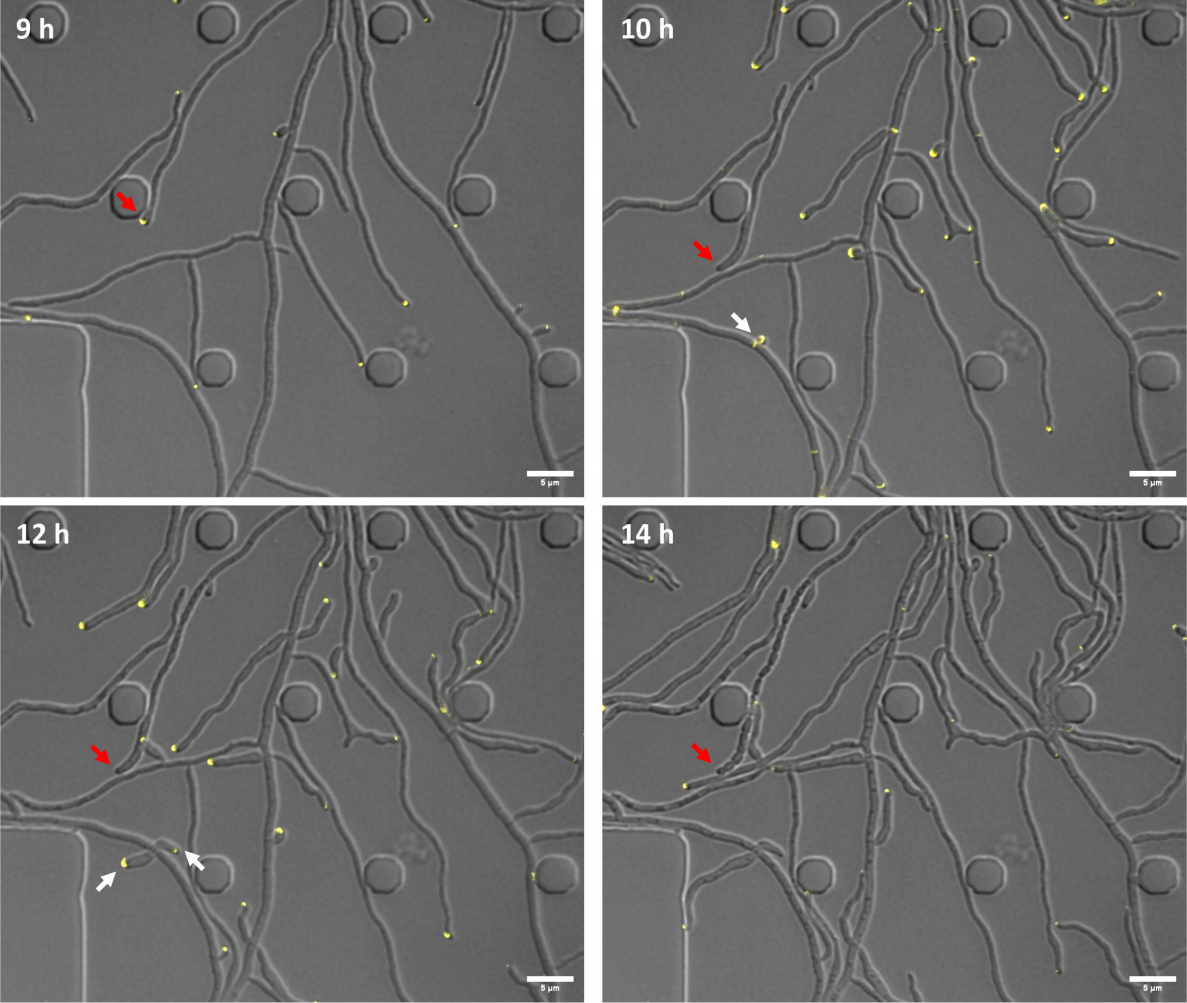
YPet-CglA localizes at tips and branching zones in growing *S. venezuelae* hyphae. Subcellular localization of fluorescently labeled CglA in *S. venezuelae* wild type expressing *ypet-cglA* from the integrative pIJ10257 vector. YPet-CglA localizes at tips of vegetative hyphae (red arrows) and at new branching points (white arrows). Snapshots from time-lapse imaging experiment at indicated time points are displayed. Scale bar: 5 µm.

To investigate the possibility that CglA is a member of the divisome or marks the position of FtsZ-ring formation, we set out to determine the subcellular localization of the protein using time-lapse fluorescence microscopy. The carboxy-terminal end of the protein is located in the extracellular space, as predicted using Protter (43). Therefore, we decided to generate a YPet-CglA protein fusion, carrying the yellow fluorescence protein at the amino-terminal end. Since the expression of *ypet-cglA* controlled by the native promoter did not yield a detectable fluorescence signal, we used pIJ10257 (Apr^R^) to express *ypet-cglA* from the constitutive ermE* promoter in *S. venezuelae*. Notably, the expression of *ypet-cglA* from pIJ10257 in the *cglA::hyg* strain fully restored the developmental defect of the mutant (Fig. S7), demonstrating that the fusion protein is functional *in vivo*. Analysis of *S. venezuelae* carrying pIJ10257-*ypet-cglA* using time-lapse fluorescence microscopy revealed that YPet-CglA specifically localizes at the tips of growing hyphae (Fig. 4; Movie S3). When following selected hyphae over time, we noticed that the apical YPet-CglA signal disappeared when the hyphae stop to extend and differentiate into chains of spores (highlighted with a red arrow in Fig. 4). Additionally, ring-like signals of YPet-CglA were occasionally detectable within vegetative hyphae, exactly where new branches appeared after some time (highlighted with a white arrow in Fig. 4). Altogether, these data demonstrate that YPet-CglA shows a distinct spatial pattern within growing hyphae and localizes in zones where new cell wall material is incorporated during growth, i.e., at the hyphal tips and branching points.

### The cell wall glycopolymer content is reduced in the *cglA* mutant

CglA belongs to the LCP family of proteins, which are well-characterized phosphotransferases catalyzing the formation of a phosphodiester bond to ligate glycopolymers to *N*-acetylglucosamine or *N*-acetylmuramic acid residues of peptidoglycan (14, 16, 17). Using structural and mutagenesis approaches, key residues for enzymatic activity of LCP domains have been determined for several enzymes of this protein family, such as TagT from *Bacillus subtilis*, LcpA from *Staphylococcus aureus,* or LcpA from *Corynebacterium glutamicum* (15, 20, 44). Amino acids sequence alignment of all *S. venezuelae* LCP domains with characterized enzymatically active reference LCP proteins shows that in CglA all residues known to be essential for activity are conserved, except K220 that appears to be located four positions downstream of the conserved locus (Fig. S8). Thus, we conclude that CglA represents an active phosphotransferase linking glycopolymers to the peptidoglycan in *S. venezuelae*. In addition, based on the conservation of active site residues, we hypothesize that Vnz_13685, Vnz_13695, and Vnz_24765 are enzymatically active, while Vnz_11555 may have lost enzymatic activity (Fig. S8).

**Fig 5:**
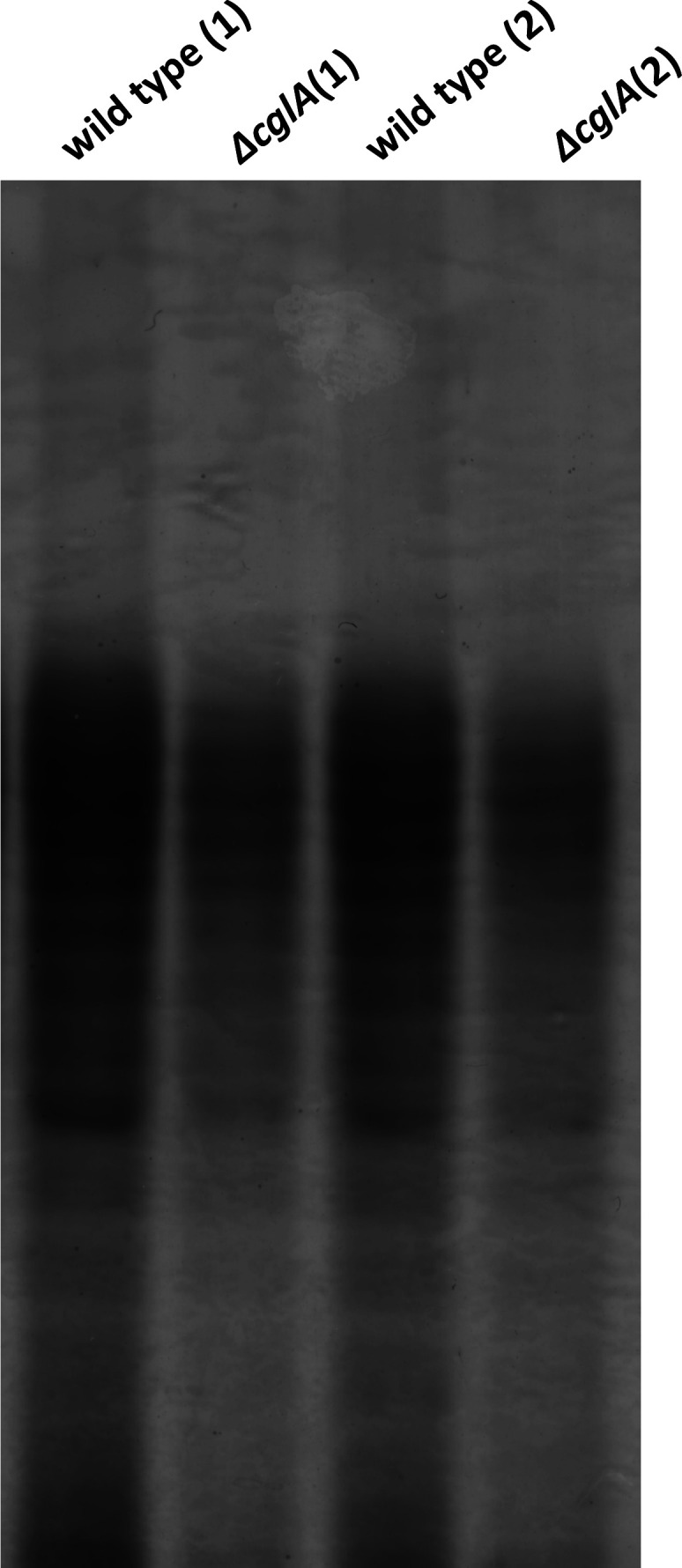
The *cglA* mutant has a reduced amount of glycopolymers in the insoluble cell wall fraction. Two independent clones of *S. venezuelae* wild type (lane 1 and 3) and of the *cglA* mutant (lane 2 and 4) were grown in liquid MYM for 16 h at 30°C. Ten milligrams of purified cell wall material was partially acid hydrolyzed to solubilize the glycopolymers which were separated by PAGE and visualized by combined alcian blue and silver staining.

To test directly for a defect in cell wall glycopolymer content in the *cglA* mutant, we extracted cell wall material comprising the peptidoglycan-glycopolymers complex out of 2.5 g (wet weight) cell pellet from wild type and the mutant strain, each represented by two independent clones. Notably, while extraction of the cell wall material from the two wild-type clones yielded 217.7 mg and 216.7 mg, respectively, the two mutant clones contained substantially less cell wall (139.4 mg and 82.9 mg) in the same biomass amount (Table 1). We then partially acid-solubilized glycopolymers with 50 mM HCl by using identical amount of cell wall material and separated them by PAGE. Visualization using alcian blue and silver staining revealed a strongly reduced amount of glycopolymers in the two clones of the *cglA* mutant, while the overall pattern of the glycopolymer fragments was very similar between wild type and the mutant strain (Fig. 5). Based on these data, we conclude that CglA has a crucial function in attaching glycopolymers to the peptidoglycan layer of *S. venezuelae*.

**TABLE 1:** The *cglA* mutant contains less cell wall material^*a*^

Strain used	Harvested biomass (g)	Extracted cell wall material (mg)
Wild type (1)	2.5	217.7
Wild type (2)	2.5	216.7
∆*cglA* (1)	2.5	139.6
∆*cglA* (2)	2.5	82.9

^*a*^ The insoluble peptidoglycan-glycopolymers complex was isolated from *S. venezuelae* wild type and *cglA* mutant in biological replicates.

## DISCUSSION

Cell shape and cell wall integrity are key factors determining bacterial vitality. Although the peptidoglycan layer is highly functionalized with glycopolymers in all monoderm bacteria, we know very little about the function of these polymers in the biology of Actinomycetota. In this study, we identified the CglA protein as a major cell wall glycopolymer ligase in the chloramphenicol producer *S. venezuelae* and demonstrated that the deletion of the gene results in a substantial reduction of the glycopolymer content in the cell wall fraction leading to cell shape defects and failures in FtsZ-rings positioning and cell septa placement (Fig. 2, 3, and 5).

Cell walls of hyphae and spores in Streptomycetes have been reported to be mainly decorated with teichulosonic acid, which is a phosphate-free polymer of up to seven repeating units composed of galactose (Gal) and the sialic acid 2-keto-3-deoxy-d-glycero-d-galacto-nononic acid (Kdn), often substituted with GlcNAc or a methyl group. As a minor component, poly(diglycosyl 1-phosphate) consisting of the repeating unit -6)-α-Gal*p*-(1→6)-α-Glc*p*NAc-(1-*P*- can also be found (19, 45). *S. venezuelae* has a set of eight LCP-domain proteins (Fig. 1A) with putative phosphotransferase activity catalyzing the formation of a phosphodiester bond to ligate glycopolymers to peptidoglycan (17). The developmental phenotype of the mutant suggests that CglA is a key glycopolymer ligase the function of which cannot be substituted by any of the other LCP domains.

Using time-lapse fluorescence imaging, we demonstrated that YPet-CglA localizes in regions of cell wall biosynthesis, i.e., at hyphal tips and branching points of growing hyphae, supporting the conclusion that the protein is involved in the coordination of cell wall biosynthesis. Deletion of *cglA* has a strong effect on a population level and causes a severe developmental defect of a *S. venezuelae* macrocolony, a phenotype, which can be fully restored by in *trans* expression of the gene controlled by the native promoter from an integrative vector (Fig. 2A). On single hypha level, we observed striking enlargement of hyphae, which were also characterized by anomalous branching phenotype and swollen tips. Likely due to the ~2-fold increased cell width of those hyphae, the assembly of regular FtsZ-YPet-rings and their correct positioning were strongly impaired, as shown using fluorescence microscopy. Consequently, enlarged hyphae of the *cglA* mutant have massive defects in cell septa placement leading to the formation of misshaped spores or mini compartments (Fig. 3), explaining why deletion of *cglA* compromises the formation of viable spores (Fig. 2B).

In support of our findings, functional LCP-domain proteins have been reported to be required for viability and normal cell morphogenesis in *Bacillus subtilis* since disruption of *tagTUV* genes causes abnormal cell bulging and loss of rod shape (9). Decreased levels of the LCP protein LcpA in *Corynebacterium glutamicum* led to a reduction in growth rate and cell viability, cell swelling, and loss of coryneform shape (20). A *Staphylococcus aureus* mutant in which all three *lcp* genes, *lcpA*, *lcpB,* and *lcpC*, have been inactivated produced a higher fraction of non-viable cells and many deformed cells with failures in cross-wall septa placement (46). While we still lack a detailed mechanistic understanding of how LCP proteins and cell wall glycopolymers contribute to cell shape maintenance, it has been demonstrated that in *B. subtilis* TagTUV proteins and peptidoglycan synthesizing enzymes form a complex with MreB proteins, the bacterial homologs of eukaryotic actin. Thus, it is believed that in rod-shaped bacteria, insertion of WTAs and deposition of peptidoglycan are coupled and guided by the cytoskeleton (9). In *Streptomyces*, two distinct multi-protein complexes coordinate cell wall biogenesis. During vegetative growth, apical extension of hyphae is directed by the polarisome with the two coiled-coil proteins DivIVA and Scy and the intermediate filament protein FilP as key components (24, 47, 48). On the other hand, synthesis of the thickened cell wall during sporulation was shown to depend on the *Streptomyces* spore wall-synthesizing complex (SSSC), which contains MreBCD, RodZ, and various penicillin-binding proteins (PBPs) and resembles the elangosome of rod-shaped bacteria (49, 50). The localization pattern of CglA suggests that the protein is a member of the polarisome; however, we did not detect any interaction between CglA and DivIVA, Scy, or FilP in our bacterial two hybrid assays (data not shown), which does not exclude that it interacts with other components of the tip organizing protein complex. Interestingly, a study using *S. coelicolor* revealed that the LCP-domain protein Sco2578 (PdtA), the *S. venezuelae* ortholog Vnz_11555, is a member of the SSSC (49). PdtA acts as a transferase attaching the minor glycopolymer poly(diglycosyl 1-phosphate) to peptidoglycan in *S. coelicolor* (19). Thus, it is also possible that CglA represents a component of SSSC in *S. venezuelae* and interacts with PBPs and/or MreB-proteins for coordinated attachment of cell wall glycopolymers to growing peptidoglycan.

**Fig 6:**
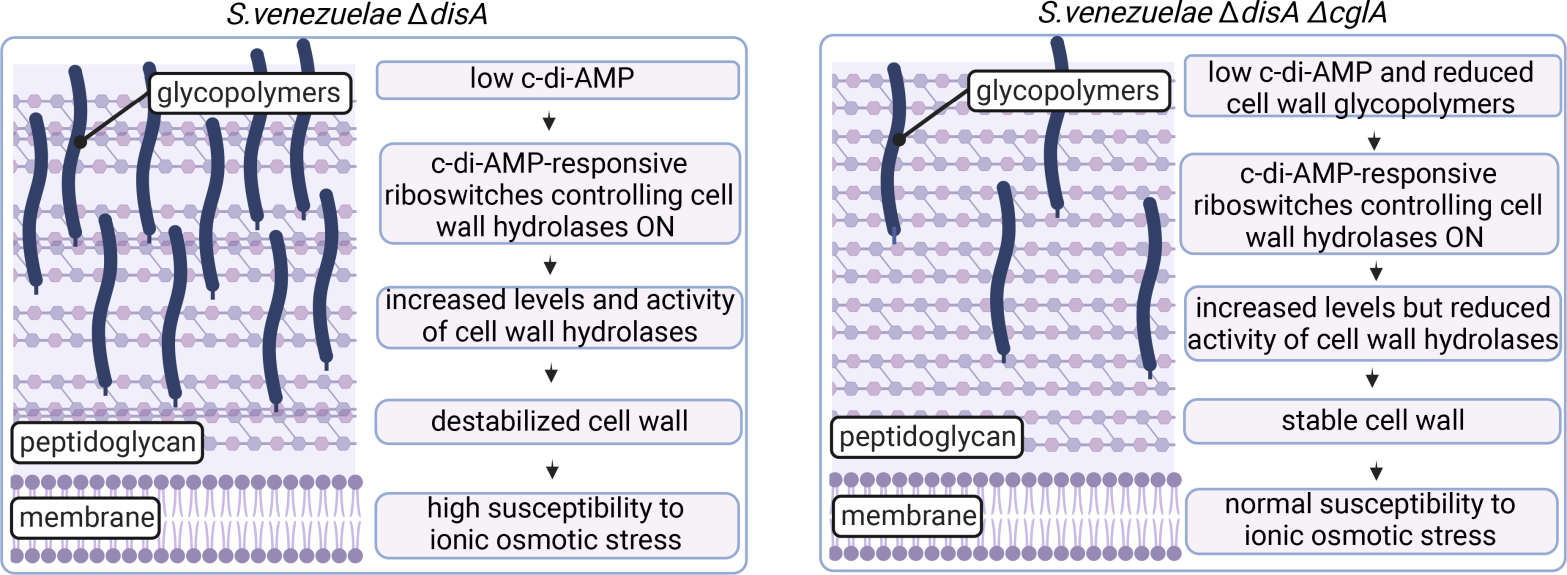
Summarizing model for the potential connection between c-di-AMP and CglA in *S. venezuelae*. Our model builds on the reported observation that localization and activity of lytic enzymes that break down peptidoglycan in bacteria is affected by wall teichoic acids (9, 10). *S. venezuelae* encodes for six cell wall hydrolases containing a c-di-AMP-responsive *ydaO*-like riboswitch in their 5′-UTR, which are known to switch into the on state at low c-di-AMP levels in the cell (53, 54). Consequently, at low levels of c-di-AMP in the *S. venezuelae disA* mutant, we expect increased expression of relevant cell wall hydrolases similar to what has been demonstrated for *rpfA* (31). The activity of those enzymes likely causes destabilization of the cell wall and, thus, increases susceptibility to osmotic stress (left panel). In contrast, reduced levels of cell wall glycopolymers in the *S. venezuelae* ∆*disA* ∆*cglA* mutant may interfere with normal activity and localization of c-di-AMP-controlled enzymes and lead to reduced cell wall hydrolysis. Overall, reduced levels of glycopolymers in the *cglA* mutant may have a stabilizing effect on the cell wall and suppress osmosensitivity of the *disA* mutant (right panel). The cell wall cartoon was created using BioRender.com.

Interestingly, we hit CglA in our screen for mutations that can compensate for the loss of c-di-AMP due to deletion of the diadenylate cyclase encoding gene *disA* (Fig. 1B; Table S1), raising the question: How are the decoration of the cell wall with glycopolymers and c-di-AMP signaling interconnected? CglA has a relatively long (~100 aa) intracellular fraction (Fig. 1A), which is important for protein function since a protein version missing this protein portion fails to complement the mutant phenotype (Fig. S3). However, according to predictions, this intracellular protein portion is disordered and flexible, which is why we find it highly unlikely that the protein is a direct c-di-AMP effector. c-di-AMP controls potassium and osmotic homeostasis and is, therefore, crucial for the regulation of cellular turgor which depends on an intact cell wall (29). Notably, the first study addressing physiological functions of c-di-AMP reports a link between c-di-AMP signaling and lipoteichoic acid biosynthesis in *Staphylococcus aureus* and demonstrates that accumulation of c-di-AMP due to the inactivation of the phosphodiesterase GdpP can compensate for the loss of lipoteichoic acid. It is thought that at high c-di-AMP, internal turgor pressure in the cell might be reduced so that lipoteichoic acid-depleted *S. aureus* cells can survive the internal pressure (51, 52). How deletion of *disA* affects ion homeostasis and turgor pressure in *Streptomyces* is not yet fully understood. However, *S. venezuelae* has six genes encoding bona fide or predicted cell wall hydrolases (*vnz_14255/rpfA*, *vnz_04045*, *vnz_36100*, *vnz_31850*, *vnz_19060*, *vnz_27285*), all of which contain a c-di-AMP responsive *ydaO*-like riboswitch in their 5′-UTR (53, 54). The *ydaO*-type riboswitch exhibits a high affinity for c-di-AMP and represents a genetic off-acting element (55). In line with that, at low c-di-AMP, the *ydaO*-like riboswitch controlling expression of *rpfA* is in the on state, leading to increased levels of RpfA (31). This likely also applies to the other five cell wall hydrolases under the control of the *ydaO*-like riboswitch. Therefore, we hypothesize that at low c-di-AMP in the *disA* mutant, increased levels of cell wall hydrolases contribute to destabilization of the cell wall and increased susceptibility of the mutant to ionic osmotic stress (Fig. 6, left panel). In contrast, reduced content of cell wall glycopolymers in the *S. venezuelae* ∆*disA* ∆*cglA* mutant may lead to reduced action of cell wall hydrolases since localization and activity of lytic enzymes that break down peptidoglycan in bacteria has been reported to be affected by wall teichoic acids (9, 10). In conclusion, we propose that deletion of *cglA* in the *disA* mutant has a stabilizing effect on the cell wall, which suppresses osmosensitivity arising at low c-di-AMP (Fig. 6, right panel), and we will challenge this model in our future work.

## MATERIALS AND METHODS

### Bacterial strains and growth conditions

All strains used in this study are listed in Table S2. *Escherichia coli* strains were grown in LB under aerobic conditions at 37°C. If needed, LB was supplemented with 50 µg/mL kanamycin (Kan), 50 µg/mL apramycin (Apr), and/or 15 µg/mL chloramphenicol (Cam). When hygromycin B (Hyg) was used, LB was replaced with LBon (LB without NaCl) and LB agar with nutrient agar (DNA; Difco Nutrient Agar) with the addition of 22 µg/mL and 16 µg/mL of Hyg, respectively. *S. venezuelae* strains (Table S2) were grown aerobically at 30°C with 180 rpm in liquid maltose-yeast extract-malt extract (MYM) medium containing 50% (vol/vol) tap water and 0.2% (vol/vol) trace element solution. For the growth of strains carrying the pIJ10257 plasmid, Hyg (22 µg/mL) was added to MYM. Spores at a final concentration of 10^6^ CFU/mL were used for inoculation.

To monitor *S. venezuelae* development, 12 µL of 2 × 10^5^ CFU/µL spores (diluted in 20% [vol/vol] glycerol) was spotted on MYM agar and macrocolonies were imaged using S9 i stereomicroscope (Leica) after 2 and 4 days of growth at 30°C. For quantification of sporulation efficiency, macrocolonies were grown for 48 h at 30°C. For each of the three biological replicates, four macrocolonies were scrapped from the plates and transferred into a 50 mL falcon tube containing 2 mL of 20% (vol/vol) glycerol. After vortexing for 1 min, the samples were incubated for 5 min at room temperature (RT). The suspension (1.5 mL) was centrifuged for 2 min at 2,000 rpm, and 1 mL of the supernatant containing the spores was filtered using a 5-µm filter. Filtrates containing the spores were diluted, and appropriate dilutions were plated on LB plates. Colonies were counted after 2 days incubation at 30°C. Sporulation efficiency in CFU/mL was plotted along with the statistical analysis using GraphPad Prism 10.

For osmostress experiments, 10 µL of serially diluted *S. venezuelae* spores (10^1^–10^4^ CFU/µL) were spotted on NA medium and NA supplemented with 0.5 M NaCl, respectively. Plates were incubated for 2 days at 30°C, and pictures were made using a Canon EOS 1300D (W) camera.

### Construction of plasmids

The oligonucleotides used for plasmid constructions are listed in Table S2. For complementation, the *cglA* gene including 234 bp upstream of the open reading frame covering the promoter region was cloned into NdeI/HindIII restriction sites of p3xFLAG vector, giving rise to a non-tagged protein. To test the effects of CglA truncation in complementation experiments, gene variants resulting in protein alterations (CglA_∆IP (amino acids 2–94), CglA_∆LCP (amino acids 180–343), and CglA_∆CC (amino acids 430–455) were cloned into p3xFLAG using Gibson assembly. Sequences of all inserts were verified by sequencing at MicroSnyth SeqLab GmbH, Göttingen. *ypet-cglA* was synthesized and sub-cloned into NdeI/HindIII restriction sites of pIJ10257 by GenScript, placing the gene fusion under the control of the constitutive ermE* promoter.

### Construction of *S. venezuelae* strains expressing *cglA* variants for complementation, *ftsZ-ypet* and *ypet-cglA*

The integrative plasmids p3xFLAG^AprR^-*cglA,* pNT101^AprR^(*ftsZ-ypet)* and pIJ10257^AprR/HygR^-*ypet-cglA* were transformed into *E. coli* ET12567/pUZ8002 for conjugation into *S. venezuelae* on SFM agar. The plates were incubated overnight at RT before overlaying with 2 mL dH_2_O containing 20 µL of 25 µg/mL nalidixic acid (Nal) and 20 µL of 50 µg/mL Apr or 20 µL of 25 µg/mL Nal and 20 µL of 50 µg/mL Hyg, respectively. Plates were incubated at 30°C until colonies appeared. Ex-conjugants were subsequently re-streaked twice on plates containing Nal and Apr or on Nal and Hyg, respectively.

### Generation of *S. venezuelae* mutants

A modified Redirect PCR targeting protocol (35) was used to generate *cglA*, *cglA*_∆482–581aa_*, vnz_13695*, *vnz_13705,* and *vnz_11555* mutants by replacing the gene of interest by the hygromycin resistance cassette. For that, *E. coli* BW25113 containing the λ-RED plasmid pIJ790 was transformed either with the cosmid Pl2_C6 (with *cglA*, *vnz_13695,* and *vnz_13705*) or with the Pl1_H1 cosmid (with *vnz_11555*) and grown in LB under aeration at 30°C. Next, BW25113/pIJ790 carrying the relevant cosmid was transformed with a PCR fragment containing the *hyg-oriT* cassette flanked by homologous regions to the desired gene locus. Successful replacement of genes of interest on cosmids by the antibiotic resistance cassette was verified by PCR using test primers (Table S2). Mutagenized cosmids were then transformed into *E. coli* ET12567/pUZ8002 for conjugation into *S. venezuelae*. Conjugation was carried out overnight at RT on SFM plates. Next day, plates were overlayed with 20 µL of 25  µg/mL Nal and 20 µL of 50  µg/mL Hyg in 2 mL dH_2_O and incubated at 30°C until colonies appeared. The desired mutants arising from a double crossing-over event were screened for a Kan-sensitive and Hyg-resistant phenotype and confirmed by PCR using test primers (Table S2).

The double mutants *cglA::hyg disA::apr; cglA*_∆482-581aa_*::hyg disA::apr* and *vnz_11555::hyg, disA::apr* were generated by transduction of the *disA::apr* (30) allele into *cglA::hyg*, *cglA*_∆482-581aa_*::hyg* or *vnz_11555::hyg* mutants using SV1 phage (56). Desired transductants were screened for Apr^R^ and Hyg^R^ phenotype and confirmed by PCR using test primers (Table S2). The double mutants *vnz_13695::hyg disA::apr* and *vnz_13705::hyg disA::apr* were generated using the Redirect PCR targeting method as described above. Correct deletions were validated by PCR and by Apr^R^ and Hyg^R^ but Kan^S^ phenotype.

### Phase-contrast and fluorescence microscopy

For visualization of the spores formed by *S. venezuelae* strains, macrocolonies were grown for 2 days at 30°C, and cover-slip imprints were made from their upper layer and imaged using phase contrast DM2000 microscope (Leica) at 100× magnification. Coverslip imprint images were used for measurements of spore length using the MicrobeJ plugin (57) in the Fiji software (58). GraphPad Prism 10 was used for plotting and statistical analysis of the data.

Time-lapse fluorescence imaging with *S. venezuelae* was conducted as described in reference 59. *S. venezuelae* strains were grown in liquid MYM (supplemented with Hyg 22 µg/mL when strain carrying the pIJ10257 derivative was used) for around 40 h at 30°C and 250 rpm. To separate the spores, mycelium was pelleted at 2,000 rpm for 1 min. Supernatants containing the spores were diluted in MYM medium 1:20 and loaded into B04A microfluidic plates (ONIX, CellASIC). Spore germination and outgrowth were induced by perfusing MYM for 4 h with subsequent media switch to spent MYM. Spent MYM was prepared from the 40-h-old *S. venezuelae* culture by filtering the growth media with a 0.2-µm filter to remove any cells. During time-lapse imaging, media flow rate at 2 psi and 30°C temperature were maintained, and images were acquired at every 15 min until sporulation was completed. Images were captured at 100× magnification with THUNDER Imager Live Cell & 3D Cell Culture coupled to a K8 GTC camera (Leica) using the CYR71010 filter at an exposure time of 80 ms and 33% of light intensity (510 nm). The movies and the snapshots prepared from time-lapse imaging were processed using the Fiji software. To determine the width of hyphae, time-lapse images obtained from the analysis of *S. venezuelae* strains carrying the pNT101 plasmid were used. For *cglA*_∆482–581aa_ with pNT101, widened hyphae (*n* = 10), and for wild type containing pNT101, random (*n* = 10) hyphae were selected, and their width was measured using the Fiji software (58).

### Transmission electron microscopy

For TEM analysis, single macrocolonies of *S. venezuelae* after 36 h of growth were cut out of an MYM agar plate and fixed in 150 mM HEPES buffer (pH 7.35), with 1.5% (vol/vol) formaldehyde and 1.5% (vol/vol) glutaraldehyde. After washing in 150 mM HEPES buffer (pH 7.35) for 2 × 6 min, and 4 × 6 min in 100 mM cacodylate buffer (pH 7.35), samples were post-fixed in 1% osmium tetroxide in cacodylate buffer (pH 7.35) for 2 h and afterward in 1% uranyl acetate in water, overnight at 4°C. Samples were dehydrated, after washing in water, 2 × 10 min, in an ascending acetone series (2 × 10 min in 70%, 2 × 10 min in 90%, and 5 × 10 min in 100%) and embedded in Epon resin (Agar 100 resin, Agar Scientific, Stansted, Essex, UK). Fifty-nanometerultrathin sections were cut, post-stained with 4% aqueous uranyl acetate and lead citrate, and analyzed at 80 kV with a FEI Morgagni 268 TEM (FEI, Eindhoven, Netherlands) with side-mounted Veleta CCD camera (Olympus Soft Imaging Solutions, Münster, Germany). Images were adapted in brightness, contrast, and shading correction.

### Extraction of cell wall material

Isolation of mycelial cell walls was performed as described in reference 60 with minor modifications. Biomass (2.5 g [wet weight]) of *S. venezuelae* wild type and *cglA* mutant was harvested by centrifugation after 16 h of growth in liquid MYM. Cell pellets were resuspended in buffer 1 (50 mM Tris-HCl, pH 7.0), followed by boiling of cells for 1.5 h in buffer 2 (5% [wt/vol] SDS, 50 mM Tris-HCl, pH 7.0) and washing steps to remove SDS. The resulting cell wall material was resuspended in buffer 3 (2% NaCl, 50 mM Tris-HCl, pH 7.0) and incubated in digestion buffer (20 mM Tris-HCl [pH 8.0], 0.5% [wt/vol] SDS) containing proteinase K with shaking at 50°C for 4 h. The suspension was sedimented by centrifugation and washed thoroughly 3–6 times with water to remove residual SDS. Resulting cell wall material was dried using a SpeedVac vacuum concentrator.

In order to detach cell wall glycopolymers from peptidoglycan and for solubilization, 10 mg of total cell wall material was hydrolyzed under moderately acidic conditions (50 mM HCl, 90°C, 15 min) according to reference 61. This treatment is harsh enough to induce cleavage of the phosphodiester bonds by which the cell wall glycopolymers are attached to the PG but leaves glycosidic and peptide bonds intact (61).

### Electrophoresis of cell wall glycopolymers

In order to separate the solubilized glycopolymer fraction containing fragments of different length, 10 µL of the hydrolyzed glycopolymer sample was mixed with 5 µL of 4× loading dye (0.1 M Tris, 0.1 M Tricine buffer, pH 8.2, with a trace of bromophenol blue) and applied to polyacrylamide gel electrophoresis. Gels (20 × 16 cm) with resolving acrylamide (30% T, 6% C) were prepared with gel buffer (3 M Tris-HCl, pH 8.5) and left for polymerization overnight at 4°C. Gel electrophoresis was performed with constant 25 mA in a PROTEAN II xi 2-D Cell with Tris-Tricine running buffer. For visualization of the fragments, the gel was stained for 2 h with alcian blue staining solution containing 5% (vol/vol) acetic acid, 40% (vol/vol) ethanol, and 0.1% (wt/vol) alcian blue. Destaining was performed with a solution composed of 10% (vol/vol) acetic acid and 20% (vol/vol) ethanol until the dye font was no longer visible, followed by silver staining with the Pierce Silver stain kit from Thermo Scientific.

## SUPPLEMENTAL MATERIAL

Supplemental material mbio.01492-24-s0001.docx Supplemental figures, movie legends, and tables.

Movie S1 mbio.01492-24-s0002.avi Localization of FtsZ-YPet in *S. venezuelae* wild type

Movie S2 mbio.01492-24-s0003.avi Localization of FtsZ-YPet in the *S. venezuelae cglA* mutant.

Movie S3 mbio.01492-24-s0004.avi Localization of YPet-CglA in *S. venezuelae* wild type.

ASM does not own the copyrights to Supplemental Material that may be linked to, or accessed through, an article. The authors have granted ASM a non-exclusive, world-wide license to publish the Supplemental Material files. Please contact the corresponding author directly for reuse.
