# Supplementary material for: Cell shape and division septa positioning in filamentous Streptomyces require a functional cell wall glycopolymer ligase CglA
Source: mBio. 2024 Sep 9;15(10):e01492-24. doi: 10.1128/mbio.01492-24 (PMC11481543; doi:10.1128/mbio.01492-24)
Supplement: Supplemental material — Supplemental figures, movie legends, and tables. [file mbio.01492-24-s0001.docx]

**Supplemental data**

# Cell shape and division septa positioning in filamentous *Streptomyces* require a functional cell wall glycopolymer ligase CglA

**Sukanya Bhowmick^1^, Ruth P. Viveros^1^, Andreas Latoscha^2#^, Fabian M. Commichau^3^, Christoph Wrede^4^, Mahmoud M. Al-Bassam^5^, and Natalia Tschowri^1*^**

*^1^Institute of Microbiology, Leibniz Universität Hannover, 30419 Hannover, Germany*

*^2^Institute of Biology/Microbiology, Humboldt-Universität zu Berlin, 10115, Berlin, Germany.*

*^#^Present address: Esencia Foods, Mollstraße 32, 10249 Berlin*

*^3^Institute of Biology, FG Molecular Microbiology 190 h, Universität Hohenheim, 70599 Stuttgart, Germany*

*^4^Institute of Functional and Applied Anatomy, Research Core Unit Electron Microscopy, Hannover Medical School, 30625 Hannover, Germany*

*^5^* *Department of Pediatrics, University of California, San Diego, La Jolla, California, United States*

**^*^**Correspondence: tschowri@ifmb.uni-hannover.de

**
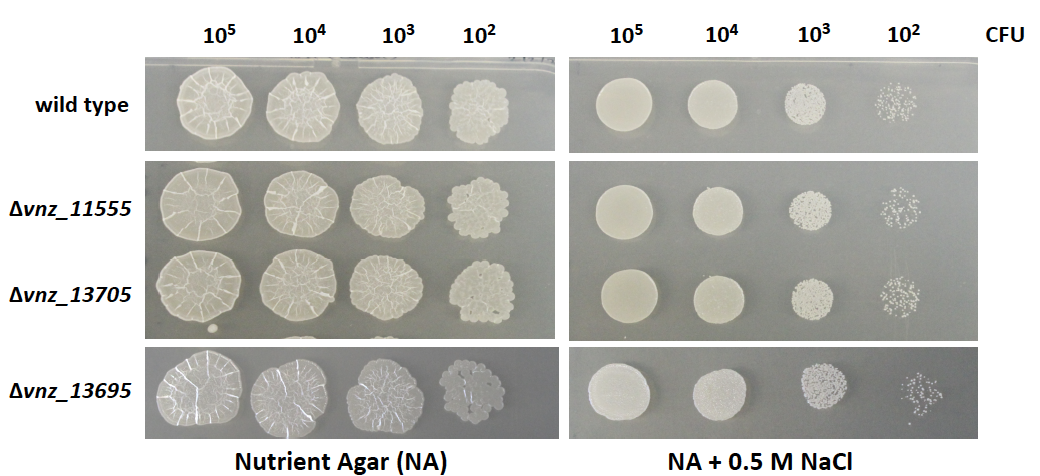
**

**Figure S1: Deletion of the LCP-LytR_C protein encoding genes *vnz_11555*, *vnz_13705* or *vnz_13695* does not compromise *S. venezuelae* growth in presence of high salt.** Serial dilutions of spores were spotted on NA with or without extra added 0.5 M NaCl and grown at 30 °C for 2 days.

**
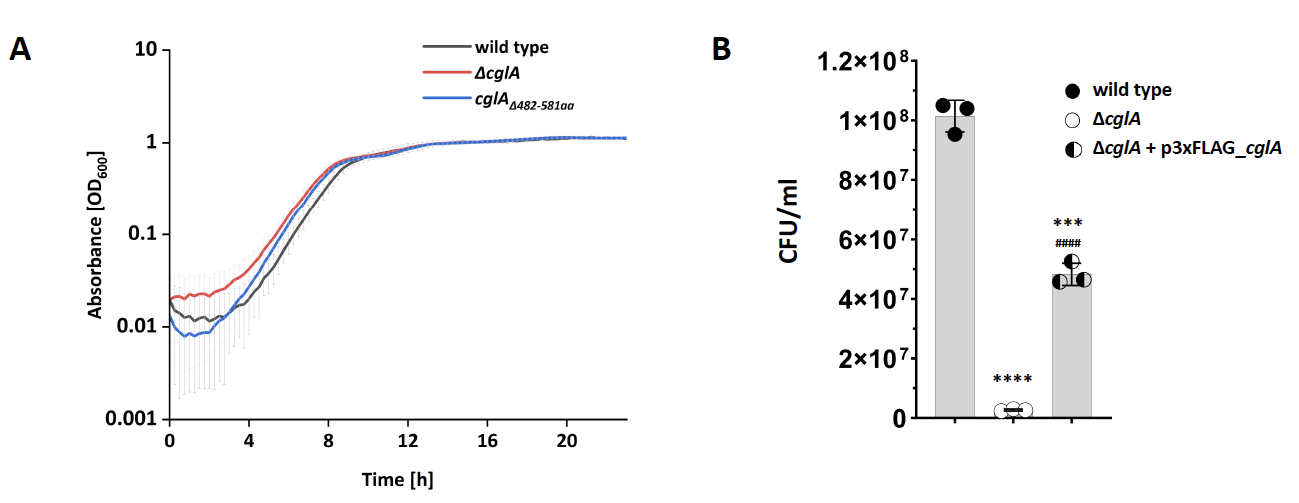
**

**Figure S2: Effects of *cglA* inactivation on growth and sporulation of *S. venezuelae* in liquid medium.** (A) 10 µl spores (10^9^ CFU/ml) of wild type, and the two mutants *cglA* and *cglA*_∆482-581aa_ were inoculated into 1 ml of liquid MYM medium in a sterile 24 well plate (Sarstedt). Strains were grown at 30 °C with shaking, and optical density was measured every 15 min for 24 h at 600 nm using SpectraMax iD5 Microplate Reader (Molecular Devices). Data from 2 independent experiments with 6 technical replicates for each set-up are plotted as mean with error bars representing standard deviation. (B) *S. venezuelae* wild type, the *cglA* mutant and the mutant strain expressing *cglA* controlled by the native promoter from the integrative p3xFLAG plasmid were grown in liquid MYM for 48 h. 2 ml of the culture were collected and centrifuged at 2,000 rpm for 2 min and then 1 ml of the supernatant (containing the spores) was filtered through a sterile 5 µm filter unit. Filtrates containing the spores were diluted and appropriate dilutions were plated on LB agar. Colonies were counted after 2 days incubation at 30°C. Data (mean CFU/ml) from 3 independent biological replicates are plotted as mean with error bars representing standard deviation, that were statistically analyzed using unpaired t-test, (***p < 0.001, ****p < 0.0001, ####p <0.0001). Comparison with wild type (*) and with ∆*cglA* (#).

**
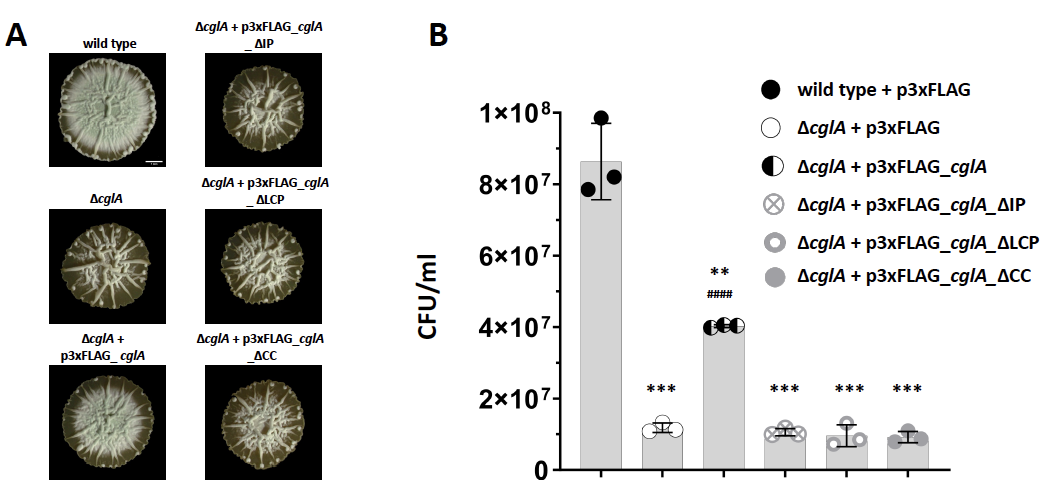
**

**Figure S3: Individual protein fractions and domains contribute to functionality or structural stability of CglA.** (A) Macrocolonies of *S. venezuelae* wild type, *cglA* mutant and mutant strain expressing various versions of *cglA* controlled by the native promoter from the integrative p3xFLAG plasmid were grown for 2 days on MYM agar. Scale bar: 1 mm. (B) Sporulation efficiency of the respective strains was determined from microcolonies grown for 48 h at 30 °C. Four colonies for each strain were scrapped from the plates and transferred into a 50 ml falcon tube containing 2 ml of 20% (v/v) glycerol. After vortexing for 1 min, the samples were incubated for 5 min at room temperature (RT). 1.5 ml of the suspension was centrifuged for 2 min at 2000 rpm and 1 ml of the supernatant containing the spores was filtered using a sterile 5 µm filter. Filtrates containing the spores were diluted and appropriate dilutions were plated on LB plates. Colonies were counted after 2 days incubation at 30°C Data (mean CFU / ml) from 3 independent biological replicates are plotted as mean with error bars representing standard deviation, that were statistically analyzed using unpaired t-test, (***p < 0.001, **p < 0.01, ####p <0.0001). Comparison with wild type + p3xFLAG (*) and with ∆*cglA* + p3xFLAG (#).

**
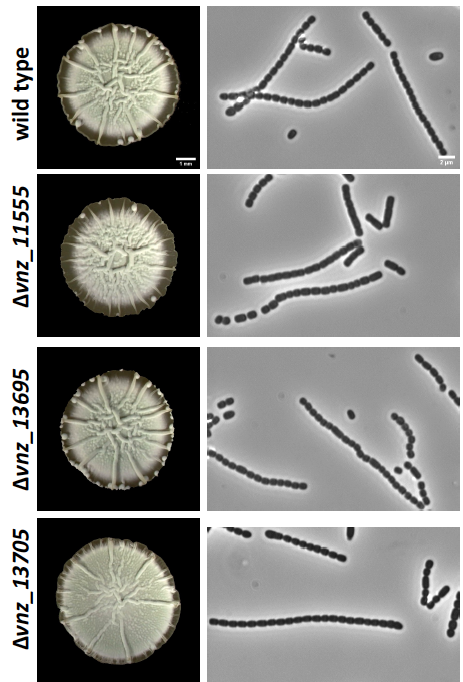
**

**Figure S4: Deletion of the LCP-LytR_C protein encoding genes *vnz_11555*, *vnz_13695* or *vnz_13705* does not affect differentiation in *S. venezuelae***. (A) Left lane: Macrocolonies of *S. venezuelae* wild type, *vnz_11555*, *vnz_13695* and *vnz_13705* mutants were grown for 2 days on MYM agar at 30 °C. Scale bar: 1 mm. Right lane: Images showing cover-slip imprints of the respective strains taken from the surface of the macrocolony. Scale bar: 2 µm.

**
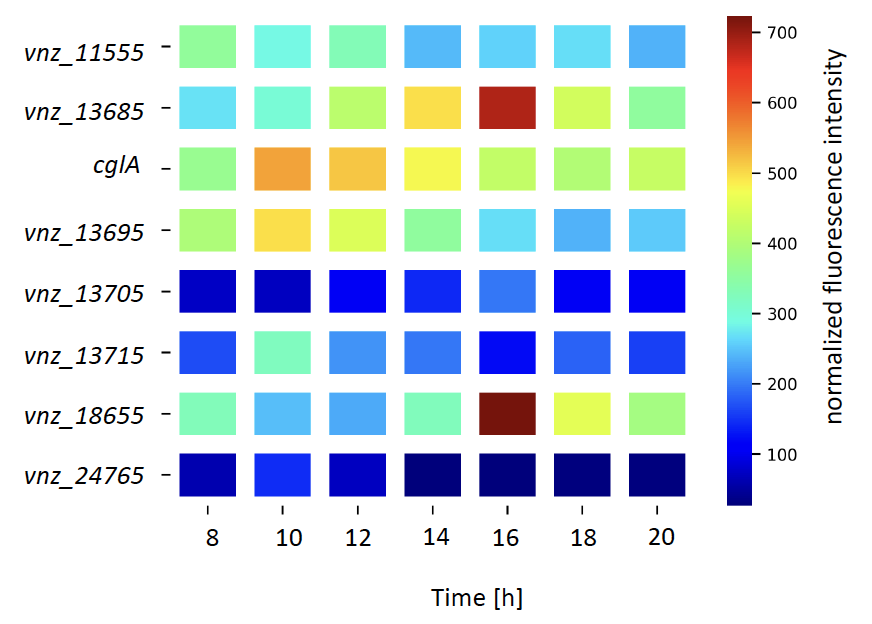
**

**Figure S5: Expression profiles of genes encoding LCP or LytR_C domain proteins during submerged sporulation of *S. venezuelae.*** Expression data were extracted from microarray analyses in (1) and normalized with quantile normalization and median polish using the RMA method as described in (2). Expression values are shown as arbitrary unit derived from the normalized fluorescence intensity. Of note, *vnz_22875* is missing in the microarray analysis from Bibb et al.

**
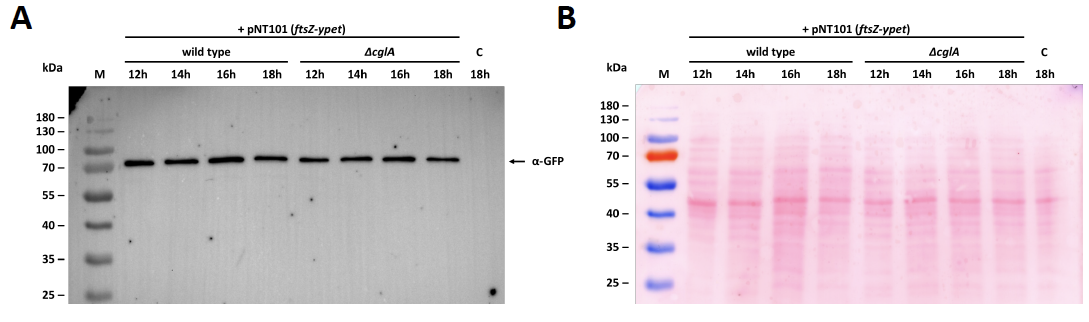
**

**Figure S6:** **Deletion of *cglA* does not affect FtsZ levels.** (A) *S. venezuelae* wild type and the *cglA* mutant, both expressing *ftsZ-ypet* controlled by the native *ftsZ* promoter from the pNT101 vector, were grown in liquid MYM at 30°C. 5 ml samples were collected after 12, 14, 16 and 18 hours of growth. A sample from *S. venezuelae* wild type without pNT101 was harvested after 18 h of growth and used as negative control (lane labelled with C). After cell lysis, total protein concentration was determined using Bradford assay (Roth) and 10 μg of total protein was separated on a 12% polyacrylamide gel. After electrophoresis, proteins were transferred to a polyvinylidene difluoride (PVDF, Roth) membrane by electroblotting using a semi-dry blotting system from Analytik Jena for 1h at 44 mA. For detection of the FtsZ-YPet protein, α-GFP antibody (Sigma) and the HRP-conjugated anti-rabbit (GE Healthcare) were used at 1:3000 and 1:10.000 dilutions, respectively. Bound secondary antibody was visualized using ClarityTM Western ECL Substrate (BioRad) and an ECL Chemocam Imager (Intas Pharmaceuticals Limited). The experiment was performed twice and a representative image is shown. (B) After blotting, the PVDF membrane was stained with Ponceau S Staining solution for 5-10 min to visualize equal sample loading.

**
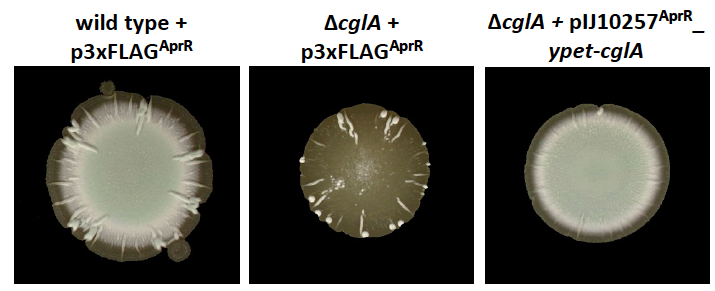
**

**Figure S7: Expression of *ypet-cglA* controlled by the constitutive *ermE** promoter from the pIJ10257 vector complements the developmental phenotype of the *cglA* mutant.** Macrocolonies of *S. venezuelae* wild type + p3xFLAG^AprR^, ∆*cglA* + p3xFLAG^AprR^ and ∆*cglA* + pIJ10257^AprR^_ *ypet-cglA* strains were grown on MYM agar containing 50 µg/ml apramycin at 30 °C for 2 days.

**
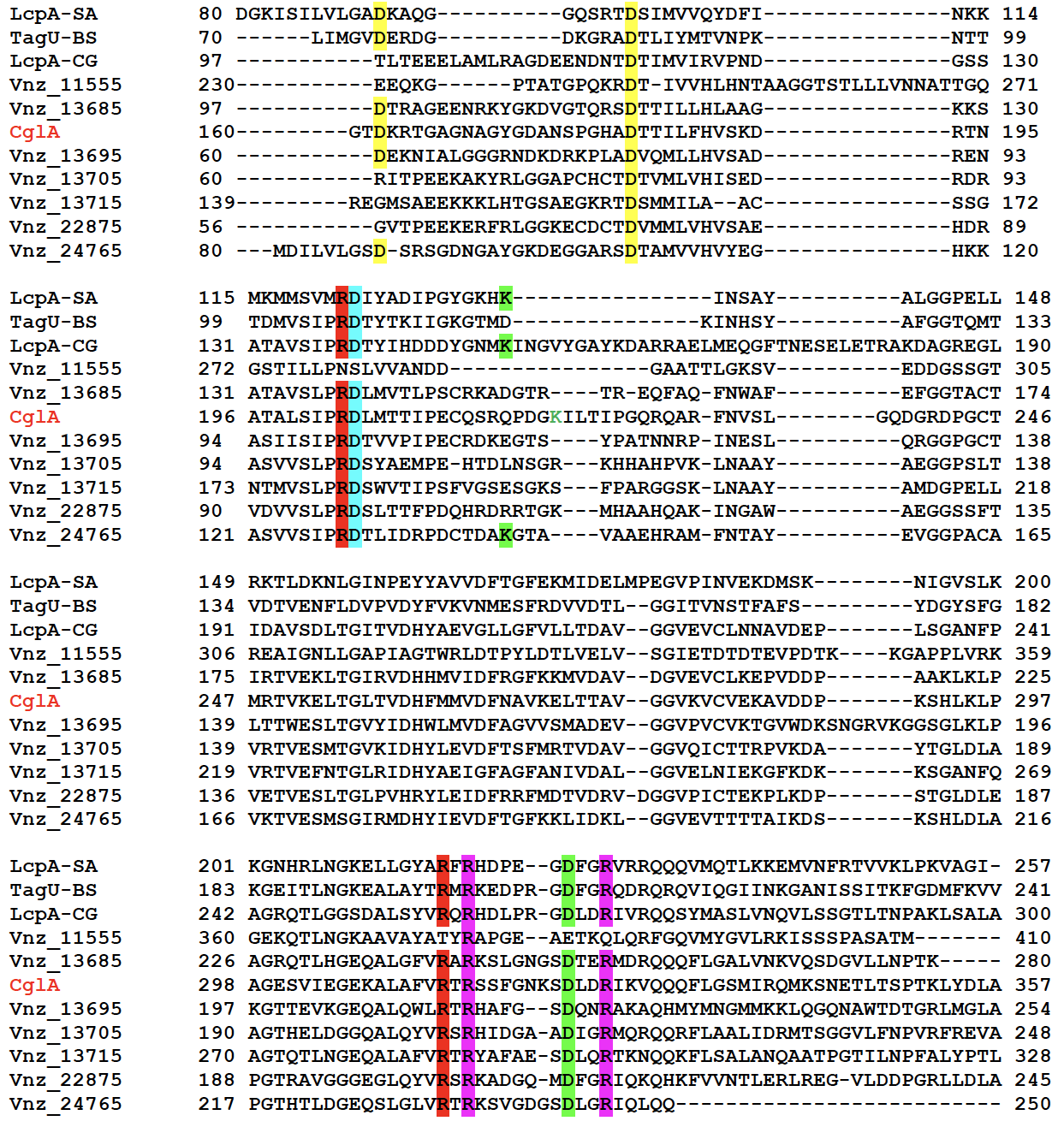
**

**Figure S8. Protein alignment between LCP domains of LcpA from *Staphylococcus aureus* (SA), TagU from *Bacillus subtilis* (BS), LcpA from *Corynebacterium glutamicum* (CG) and LCP domains from *S. venezuelae* proteins.** Colour code: Mg^2+^ coordination, general base and acid, activation of general base, peptidoglycan binding, donor substrate binding. Assignment of the residues is based on structural or mutagenesis analysis of LCP enzymes in (3), (4), (5, 6).

**Legends to Movies S1-S3:**

**Movie S1:** Time-lapse fluorescence microscopy movie showing the localization of FtsZ-YPet in *S. venezuelae* wild type (for details see legend to Fig. 3A and main text).

**Movie S2:** Time-lapse fluorescence microscopy movie showing the localization of FtsZ-YPet in the *S. venezuelae* *cglA* mutant (for details see legend to Fig. 3B and main text).

**Movie S3:** Time-lapse fluorescence microscopy movie showing the localization of YPet-CglA in *S. venezuelae* wild type (for details see legend to Fig. 4 and main text).

**Table S1. Gene mutations identified in osmoresistant suppressor mutants of *S. venezuelae* ∆*disA*.** Purified genomic DNA was paired end sequenced (Illumina NovaSeq 2 × 150 bp sequencing; GENEWIZ Germany GmbH, Leipzig). The reads were mapped onto the *S. venezuelae* NRRL B-65442 reference genome and plasmid CP018074 and CP018075, respectively, from GenBank (7) as previously described (8) using the Geneious Prime software package (9).

| **Suppressor** | **Gene / Gene mutation** | **Consequence in protein** | **Predicted function (uniprot.org)** |
| --- | --- | --- | --- |
| *∆disA*-S1 | *vnz_13690* / G1446A | Trp 482 → Stop  protein truncation | cell envelope-associated transcriptional attenuator LytR-CpsA-Psr |
|  | *vnz_14455* / C846 insertion | frameshift | nucleic acid/nucleotide deaminase of polymorphic system toxin |
| *∆disA*-S2 | *vnz_10870* / deletion after 134 bp | protein truncation | putative integral membrane protein |
| *∆disA*-S10 | *vnz_00680* / A317G | Tyr 106 → Cys  mutation | putative integral membrane protein |
|  | *vnz_33285* / T1292C | Leu 431→ Pro | uncharacterized protein |
| *∆disA*-S12 | *vnz_26710* / C1125G; T1127C; A1138C; C1140G | Val 376 → Ala;  Ile 380 → Leu; | hybrid sensor kinase / response regulator, OsaA |
| *∆disA*-S14 | *vnz_21475* / C1057T | Arg 353 → Cys | DNA-directed RNA polymerase subunit beta’ / *rpoC* |

**Table S2. Strains, plasmids and oligonucleotides used in this study**

| **Strains / NT Strain collection number** | **Genotype or comments** | **Source or reference** |
| --- | --- | --- |
| ***S. venezuelae*** | | |
| NRRL B-65442 | Wild type | (NCBI Reference Sequence: NZ_CP018074.1) |
| 552 | *S. venezuelae vnz_15820 (disA)*::*apr*; Apr^R^ | (10) |
| 670 | *S. venezuelae cglA*::*hyg-oriT*; Hyg^R^ | This study |
| 671 | *S. venezuelae cglA*_∆482-581aa_::*hyg-oriT*; Hyg^R^ | This study |
| 672 | *S. venezuelae cglA*::*hyg-oriT* *disA*::*apr*; Hyg^R^, Apr^R^ | This study |
| 673 | *S. venezuelae cglA*_∆482-581aa_::*hyg-oriT* *disA*::*apr*; Hyg^R^, Apr^R^ | This study |
| 692 | *S. venezuelae cglA*::*hyg-oriT* *attB_ΦBT1_*:: p3xFLAG; Hyg^R^, Apr^R^ | This study |
| 821 | *S. venezuelae* wild type *attB_ΦBT1_*:: p3xFLAG; Apr^R^ | This study |
| 694 | *S.venezuelae cglA::hyg-oriT attB_ΦBT1_::*p3xFLAG *_cglA;* Hyg^R^*,* Apr^R^ | This study |
| 695 | *S.venezuelae* *cglA*_∆482-581aa_::*hyg-oriT* *attB_ΦBT1_*::p3xFLAG _ *cglA*; Hyg^R^, Apr^R^ | This study |
| 807 | *S. venezuelae cglA::hyg-oriT* *attB_ΦBT1_*:: p3xFLAG _ *cglA* _∆IP; Hyg^R^, Apr^R^ | This study |
| 808 | *S. venezuelae cglA::hyg-oriT* *attB_ΦBT1_*:: p3xFLAG_ *cglA* _∆LCP; Hyg^R^, Apr^R^ | This study |
| 809 | *S. venezuelae* *cglA::hyg-oriT* *attB_ΦBT1_*:: p3xFLAG_ *cglA* _∆CC; Hyg^R^,Apr^R^ | This study |
| 852 | *S. venezuelae vnz_11555::hyg-oriT*; Hyg^R^ | This study |
| 853 | *S. venezuelae vnz11555::hyg-oriT* *disA*::*apr*; Hyg^R^,Apr^R^ | This study |
| 986 | *S. venezuelae vnz_13695::hyg-oriT*; Hyg^R^ | This study |
| 987 | *S. venezuelae vnz_13695::hyg-oriT* *disA*::*apr*; Hyg^R^, Apr^R^ | This study |
| 988 | *S. venezuelae vnz_13705*::*hyg-oriT*; Hyg^R^ | This study |
| 989 | *S. venezuelae vnz_13705::hyg-oriT* *disA*::*apr*; Hyg^R^,Apr^R^ | This study |
| 994 | *S.venezuelae* wild type *attB_ΦBT1_*:: pIJ10257 *ypet* _*cglA*; Hyg^R^ | This study |
| 990 | *S. venezuelae cglA::hyg-oriT* *attB_ΦBT1_*:: pIJ10257_ *ypet* -*cglA*; Hyg^R^, Apr^R^ | This study |
| 706 | *S. venezuelae* wild type *attB_ΦBT1_::P_ftsZ_-ftsZ-ypet*; Apr^R^ | This study |
| 707 | *S. venezuelae cglA::hyg-oriT,* *P_ftsZ_-ftsZ-ypet*; Hyg^R^, Apr^R^ | This study |
| 708 | *S. venezuelae cglA_∆482-581aa_::hyg-oriT*, *attB_ΦBT1_::* *P_ftsZ_-ftsZ-ypet*; Hyg^R,^, Apr^R^ | This study |
| ***E. coli*** | | |
| DH5α | *fhuA2 lac(del)U169 phoA glnV44 Φ80' lacZ(del)M15 gyrA96 recA1 relA1 endA1 thi-1 hsdR17* | Thermo Fisher Scientific |
| ET12567/pUZ8002 | *dam*, *dcm*, *hsd*; Kan^R^, Cm^R^ | (11) |
| BW25113/pIJ790 | (Δ(*araD-araB*)*567*, Δ*lacZ4787*(::*rrnB-4*), *lacIp-4000*(lacI^Q^), λ-, *rpoS369*(Am), *rph-1*, Δ(*rhaD-rhaB*)568, *hsdR514*; Cm^R^ | (12) |
| **Plasmids / NT plasmid collection or strain collection number** | | |
| pIJ773 | Plasmid template for amplification of the *apr-oriT* cassette for ‘Redirect’ PCR-targeting; Apr^R^ | (13) |
| pIJ790 | Modified λRED recombination plasmid [*oriR101*] [*repA101*(ts)] *araBp-gam-be-exo*; Cm^R^ | (13) |
| pIJ10257 | Plasmid integrating at the ϕBT1 *attB* attachment site containing the constitutive *ermE** promoter, Hyg^R^ | (14) |
| pUZ8002 | RP4 derivative with defective oriT; Kan^R^ | (11) |
| p3xFLAG | pIJ10770 derivative containing 3xFLAG sequence downstream of MCS; Hyg^R^ | (2) |
| p3xFLAG^AprR^ (pNT106 / 701) | Exchange of Hyg cassette to Apr cassette on p3xFLAG using λRED recombination; Apr^R^ | This study |
| p3xFLAG^AprR^ _*cglA* (pNT107 / 702) | Expression of *cglA* (*vnz_13690)* from the ϕBT1 *attB* attachment site controlled by the native promoter; Apr^R^ | This study |
| p3xFLAG^AprR^_*cglA*_∆IP (pNT108 / 822) | Expression of *cglA* (*vnz_13690)* without the intracellular portion (amino acids 2-94) from the ϕBT1 *attB* attachment site controlled by the native promoter; Apr^R^ | This study |
| p3xFLAG^AprR^_*cglA*_∆LCP (pNT109 / 823) | Expression of *cglA* (*vnz_13690)* without the LCP domain (amino acids 180-343) from the ϕBT1 *attB* attachment site controlled by the native promoter; Apr^R^ | This study |
| p3xFLAG^AprR^_*cglA*_∆CC (pNT110 / 824) | Expression of *cglA* (*vnz_13690)* without the coiled-coil region (amino acids 430-455) from the ϕBT1 *attB* attachment site controlled by the native promoter; Apr^R^ | This study |
| pIJ10257_*ypet*-*cglA* (pNT319 / 983) | Expression of *ypet*-*cglA* (*vnz_13690)* from the ϕBT1 *attB* attachment site controlled by the constitutive *ermE** promoter, Hyg^R^ | This study, generated by GenScript |
| pIJ10257^AprR^_*ypet*-*cglA* (pNT321 / 985) | Exchange of Hyg cassette on pNT319 to Apr cassette using λRED recombination. Expression of *ypet*-*cglA* (*vnz_13690)* from the ϕBT1 attB attachment site controlled by the constitutive *ermE** promoter, Apr^R^ | This study |
| pSS05 (*ftsZ-ypet)*/ 102 | Expression of *ftsZ-ypet* from the ϕBT1 *attB* attachment site controlled by the native *ftsZ* promoter of *S. venezuelae*; Hyg^R^ | (15) |
| pNT101 (*ftsZ-ypet)*/ 715 | Exchange of Hyg cassette on pSS05 to Apr cassette using λRED recombination. Expression of *ftsZ-ypet* from the ϕBT1 *attB* attachment site controlled by the native *ftsZ* promoter of *S. venezuelae*; Apr^R^ | This study |
| **Oligonucleotides used for generation and testing of mutants (**sequence homologous to antibiotic resistance cassettes in italics) | | |
| **Name** | **Sequence 5´-3´** | |
| vnz_13690 fwd | CGCCTCGCGTGGCACCGACGGAGGACTCGAGGAACCGTG *ATTCCGGGGATCCGTCGACC* | |
| vnz_13690 rev | TCAGTCAGGAGCGAGCCAAGTCCCCGTTTTCACGGGCTA *TGTAGGCTGGAGCTGCTTC* | |
| vnz_13690 fwd trunc | AGCGGGATCCAGGGCGCGGCCCAGTCGACCATCAACTGA*ATTCCGGGGATCCGTCGACC* | |
| vnz_13690 fwd t | AATTTGTCACGCACGTCGCC | |
| vnz_13690 rev t | TGGCTTCACCGGCGCCGTAC | |
| disA_test_f | GTGGTTCACTCACGCCGCATGAACGGTTC | |
| disA_test_r | GGCACGTACCTGGTGGAGGCGAAGGTG | |
| vnz_11555 fwd | TGTACCGCGGCGAGTGAGCTTCGGGGAGGGCCACCGGTG *ATTCCGGGGATCCGTCGACC* | |
| vnz_11555 rev | CCGCCGACGCCCCCGCCAACGTTTTCCCGGGCGCCGCTA *TGTAGGCTGGAGCTGCTTC* | |
| vnz_11555 test_fwd | GACGACGGACTGCCCAAGGGC | |
| vnz_11555 test_rev | GACAGCACGTCGCTGACGTCG | |
| vnz_13695 fwd | GGCAAGCGCAAGGCGCTGCGCTGGGTGGCGATCACCCTG *ATTCCGGGGATCCGTCGACC* | |
| vnz_13695 rev | GCGGGGACCCGGCCATCAGGCGTCTGCGGGAACGGTTCA *TGTAGGCTGGAGCTGCTTC* | |
| vnz_13695 _fwd test | AGGTACAAGCTGACAAGCAG | |
| vnz_13695 _rev test | TACCACCAGGGCTCCATCAAC | |
| vnz_13705 fwd | CCGGTCCGCAGACGGCGCGGACGCCCACGGTGGGGGATG *ATTCCGGGGATCCGTCGACC* | |
| vnz_13705 rev | ACGGTCACGGGCACACCACCCCGCCCCCGGTGACGGTCA *TGTAGGCTGGAGCTGCTTC* | |
| vnz_13705 test _fwd | TACCGCGAACGGGGGACAGC | |
| vnz_13705 test _rev | CGCAACCAGGAGGCCCAGAT | |
| **Oligonucleotides used for plasmid construction and cloning** | | |
| **Oligonucleotides for exchanging the Hyg to Apr resistance cassette in p3xFLAG/ pIJ10257-*ypet-cglA* and test primers** | | |
| p3Flag_AprA_H1_P1 | GAATTAAGCCGGCCCGTACCCTGTGAATAGAGGTCCGCT *GTGCAATACGAATGGCGAAAAG* | |
| p3Flag_AprA_H3_P3 | GGGGCGGTGTCCGGCGGCCCCCAGAGGAACTGCGCCAGT *TTATGAGCTCAGCCAATCGAC* | |
| p3Flag_AprA_test_fw_2 | CTGCGGTGATAAATTATCTCTG | |
| p3Flag_AprA_test_rev_2 | CAGGTCGACTCTAGCTAGCTG | |
| **Oligonucleotides used for cloning of *vnz_13690 (cglA)* into p3xFLAG for complementation analysis and test primers for verification of successful cloning in p3xFLAG** | | |
| 13690_HindIII_fwd | CCCAAGCTTTAAAGGGCACACCAAGGGGAG | |
| 13690_NdeI_rev | GGGAATTCCATATGCTACTTGGCGCACACCTGCTTG | |
| pFLAGpSS170_seqfw | GCTCAGTGGAACGAAAACT | |
| pFLAGpSS170_seqrev | CTGATGTGCTCAGTATCACC | |
| **Oligonucleotides used for the verification of the Hyg^R^ to Apr^R^ exchange in pIJ10257-*ypet-cglA*** | | |
| pIJ10257_AprA_testF | GGTCTGACGCTCAGTGGAACG | |
| pIJ10257_AprA_testR | TTATCTCTGGCGGTGTTGACATAAATACC | |
| **Oligonucleotides used for deletion of different domains in Vnz_13690 (CglA) by Gibson Assembly** | | |
| **Oligonucleotides used for the generation of p3xFLAG-*vnz_13690 (cglA)*_∆IP** | | |
| 13690_del_IP_LF1 | *AGCTCCATCAGCAAAAGGGGATGATAAGTTTATCAAGCTT* TAAAGGGCACACCAAGGGGAG | |
| 13690_del_IP_LR1 | *ACAGCAGGACCTTCTTCTTGCGGGACGCGCCCTGCTTGCG* CACGGTTCCTCGAGTCCTCC | |
| 13690_del_IP_RF2 | CGCAAGCAGGGCGCGTCCCGC | |
| 13690_del_IP_RR2 | *ATGTACACCTAGGCTTAAGTCGCGAATCGATGATCATATG* CTACTTGGCGCACACCTGCTTGTCAGCTTGT | |
| **Oligonucleotides used for the generation of p3xFLAG-*vnz_13690 (cglA)*_∆LCP** | | |
| 13690_del_LCP_LF1 | *AGCTCCATCAGCAAAAGGGGATGATAAGTTTATCAAGCTT* TAAAGGGCACACCAAGGGGAGGGTTGC | |
| 13690_del_LCP_LR1 | *CGAGGTCGTACAGCTTCGTCGGGCTGGTGAGCGTCTCGTT* GCCGGGGCTGTTGGCGTCGC | |
| 13690_del_LCP_RF2 | AACGAGACGCTCACCAGCCC | |
| 13690_del_LCP_RR2 | *ATGTACACCTAGGCTTAAGTCGCGAATCGATGATCATATG* CTACTTGGCGCACACCTGCTTG | |
| **Oligonucleotides used for the generation of p3xFLAG -*vnz_13690 (cglA)*_∆CC** | | |
| 13690_del_CC_LF1 | *AGCTCCATCAGCAAAAGGGGATGATAAGTTTATCAAGCTT* TAAAGGGCACACCAAGGGGAG | |
| 13690_del_CC_LR1 | *CGTTGAAGACGTCGACGCGCACCTCGGCGGCGGTGGCCCG* CGAGGTGTCGGCCTTCATCG | |
| 13690_del_CC_RF2 | CGGGCCACCGCCGCCGAGGT | |
| 13690_del_CC_RR2 | *ATGTACACCTAGGCTTAAGTCGCGAATCGATGATCATATG* CTACTTGGCGCACACCTGCTTGTCAGCTTGTACCTTCTCG | |

**Supplemental References**

1. Bibb MJ, Domonkos A, Chandra G, Buttner MJ. 2012. Expression of the chaplin and rodlin hydrophobic sheath proteins in *Streptomyces venezuelae* is controlled by sigma(BldN) and a cognate anti-sigma factor, RsbN. *Mol Microbiol* 84:1033-49.

2. Al-Bassam MM, Haist J, Neumann SA, Lindenberg S, Tschowri N. 2018. Expression Patterns, Genomic Conservation and Input Into Developmental Regulation of the GGDEF/EAL/HD-GYP Domain Proteins in *Streptomyces*. *Front Microbiol* 9:2524.

3. Schaefer K, Owens TW, Kahne D, Walker S. 2018. Substrate Preferences Establish the Order of Cell Wall Assembly in *Staphylococcus aureus*. *J Am Chem Soc* 140:2442-2445.

4. Kawai Y, Marles-Wright J, Cleverley RM, Emmins R, Ishikawa S, Kuwano M, Heinz N, Bui NK, Hoyland CN, Ogasawara N, Lewis RJ, Vollmer W, Daniel RA, Errington J. 2011. A widespread family of bacterial cell wall assembly proteins. *EMBO J* 30:4931-41.

5. Baumgart M, Schubert K, Bramkamp M, Frunzke J. 2016. Impact of LytR-CpsA-Psr Proteins on Cell Wall Biosynthesis in *Corynebacterium glutamicum*. *J Bacteriol* 198:3045-3059.

6. Li FKK, Rosell FI, Gale RT, Simorre JP, Brown ED, Strynadka NCJ. 2020. Crystallographic analysis of *Staphylococcus aureus* LcpA, the primary wall teichoic acid ligase. *J Biol Chem* 295:2629-2639.

7. Gomez-Escribano JP, Holmes NA, Schlimpert S, Bibb MJ, Chandra G, Wilkinson B, Buttner MJ, Bibb MJ. 2021. *Streptomyces venezuelae* NRRL B-65442: genome sequence of a model strain used to study morphological differentiation in filamentous actinobacteria. *J Ind Microbiol Biotechnol* 48.

8. Widderich N, Rodrigues CD, Commichau FM, Fischer KE, Ramirez-Guadiana FH, Rudner DZ, Bremer E. 2016. Salt-sensitivity of sigma(H) and Spo0A prevents sporulation of *Bacillus subtilis* at high osmolarity avoiding death during cellular differentiation. *Mol Microbiol* 100:108-24.

9. Kearse M, Moir R, Wilson A, Stones-Havas S, Cheung M, Sturrock S, Buxton S, Cooper A, Markowitz S, Duran C, Thierer T, Ashton B, Meintjes P, Drummond A. 2012. Geneious Basic: an integrated and extendable desktop software platform for the organization and analysis of sequence data. *Bioinformatics* 28:1647-9.

10. Latoscha A, Drexler DJ, Al-Bassam MM, Bandera AM, Kaever V, Findlay KC, Witte G, Tschowri N. 2020. c-di-AMP hydrolysis by the phosphodiesterase AtaC promotes differentiation of multicellular bacteria. *Proc Natl Acad Sci U S A* 117:7392-7400.

11. Paget MS, Chamberlin L, Atrih A, Foster SJ, Buttner MJ. 1999. Evidence that the extracytoplasmic function sigma factor sigmaE is required for normal cell wall structure in *Streptomyces coelicolor* A3(2). *J Bacteriol* 181:204-11.

12. Datsenko KA, Wanner BL. 2000. One-step inactivation of chromosomal genes in *Escherichia coli* K-12 using PCR products. *Proc Natl Acad Sci U S A* 97:6640-5.

13. Gust B, Challis GL, Fowler K, Kieser T, Chater KF. 2003. PCR-targeted *Streptomyces* gene replacement identifies a protein domain needed for biosynthesis of the sesquiterpene soil odor geosmin. *Proc Natl Acad Sci U S A* 100:1541-6.

14. Hong HJ, Hutchings MI, Hill LM, Buttner MJ. 2005. The role of the novel Fem protein VanK in vancomycin resistance in *Streptomyces coelicolor*. *J Biol Chem* 280:13055-61.

15. Schlimpert S, Wasserstrom S, Chandra G, Bibb MJ, Findlay KC, Flärdh K, Buttner MJ. 2017. Two dynamin-like proteins stabilize FtsZ rings during *Streptomyces* sporulation. *Proc Natl Acad Sci U S A* 114:E6176-E6183.
